# Ontogeny of the Skeleton of *Leporinus oliveirai* (Characiformes, Anotomidae)

**DOI:** 10.1002/jmor.70053

**Published:** 2025-05-21

**Authors:** Mariana Pascoal Boaretto, Marcos Venturieri, José Luís Olivan Birindelli

**Affiliations:** ^1^ Programa de Pós‐Graduação em Ciências Biológicas, Departamento de Biologia Animal e Vegetal Universidade Estadual de Londrina Londrina Paraná Brazil; ^2^ Piscicultura Tanganyika Aquiraz Ceará Brazil; ^3^ Laboratório de Ictiologia, Museu de Zoologia da Universidade Estadual de Londrina Londrina Paraná Brazil

**Keywords:** anostomidae, comparative anatomy, development, ontogeny

## Abstract

The skeleton is a rich source of characters used in phylogenetic studies of teleost fishes. However, the development of bones was studied in a limited number of species and few studies have been published describing the ontogeny of the Characiformes skeleton. We provide the first complete description of the skeleton ontogeny of an anostomid, *Leporinus oliveirai*, based on specimens bred in captivity and sampling the first 60 days post‐hatching, with sizes ranging from 3.8 mm (notochord length, NL) to 33.2 mm (standard length, SL). Sixty‐three specimens were cleared and double stained and subsequently dissected and photographed. The developmental sequence of 141 bony elements is documented. Photography of all anatomical complexes is presented during the development. The first bone to develop is the cleithrum (5.1 mm NL) and the last ones are infraorbitals 4, 5 and 6, extrascapular and sclerotic bones (27.2‐29.7 mm SL), similar to what is observed in the development of other characiforms. The main discoveries are numerous conical teeth on the premaxilla and dentary from 5.1 mm NL to 10.4 mm SL that are replaced with three or four large incisiform multicuspid teeth, that become unicuspid in juveniles and adults. The infraorbitals 4 and 5, seen only in juveniles, develop fused, a condition that is in contrast to most anostomids. The autopalatine cartilage begins its development straight, becoming curved during development. The developmental sequence is compared with other Characiformes and the unique characteristics of Anostomidae are discussed concerning the phylogenetic relationships among the family members.

## Graphical abstract

In this study, we provide the first complete description of the skeleton ontogeny of an anostomid. The developmental sequence of 141 bony elements is described and illustrated. The main discoveries are numerous conical teeth on the premaxilla and dentary that are replaced with three or four large incisiform multicuspid teeth, that become unicuspid in juveniles and adults. The infraorbitals 4 and 5 develop fused, a condition that is the opposite in most anostomids. The autopalatine cartilage begins its development straight, becoming curved during development.

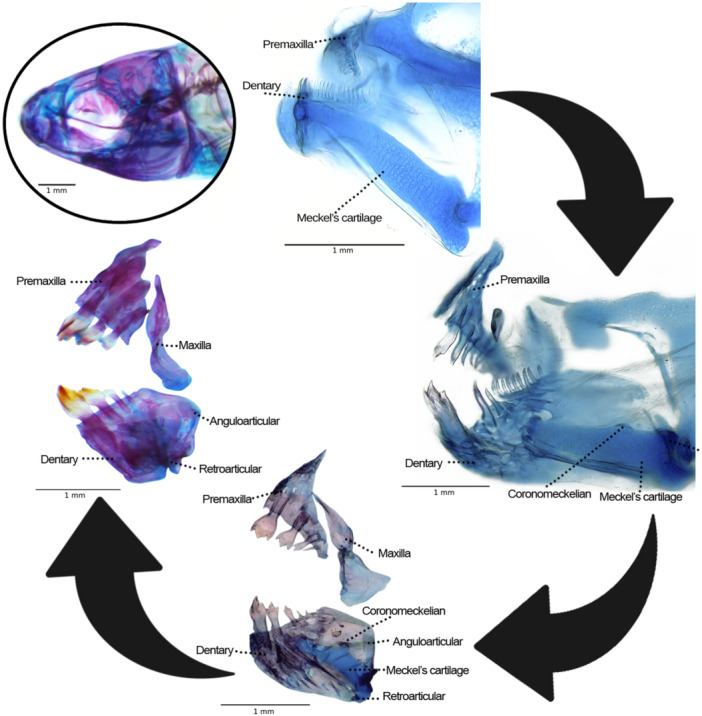

## 1 Introduction

The skeleton is one of the most studied anatomical systems and a rich source of characters to understand the evolution of teleost fishes (Mattox et al. 2014; Marinho 2022). Even though bones have been essential for phylogenetic studies, most research (Vari 1983; Sidlauskas and Vari 2008) has focused on describing the skull of adult specimens, overlooking the development of those bones. The detailed study of the skeletal ontogeny can offer important data to clarify loss or fusion of bones (Kubicek 2022) and to uncover the ancestral state of a phylogenetic character (Walter 2012; Mattox et al. 2014), knowledge that can be essential to uncover relationships among taxa (Ahlstrom and Moser 1976). The sequence of bone formation also brings to light the functional demand of the larvae that results in certain structures developing first, such as the bones related to feeding and breathing, which are highly associated with the larvae's ability to survive (Vandewalle et al. 2005; Marinho 2022).

In South America, Characiformes are one of the most abundant group of fishes (Nelson et al. 2016). Understanding the evolution of Neotropical fishes has been a huge challenge for ichthyologists (Toledo‐Piza et al. 2024). Ontogenetic studies on South‐American fishes often focus on detailing the embryogenesis and external morphology of the first days of life of the larvae, mostly giving insightful data for larvae identification (Nakatani et al. 2001; Walter 2012; Sousa. 2014; Perrotti et al. 2018; Rocha et al. 2019; Santos et al. 2020; Santos et al. 2022; Santos et al. 2024).

However, the development of the skeleton is rarely studied. Mattox et al. (2014) made a detailed description of *Salminus brasiliensis* (Cuvier 1816) and, more recently, Marinho (2022) described the ontogeny of *Makunaima pittieri* (Eigenmann 1920). Other notable studies encompassing Characiformes focused on the cranium. Vandewalle et al. (2005) described the head development of *Brycon moorei* Steindachner 1878; Walter (2012) studied the skull ontogeny of *Bario sanctaefilomenae* (Steindachner 1907), and Carvalho and Vari (2015) described the development of the splanchnocranium of *Prochilodus argenteus* Spix and Agassiz 1829.

Anostomidae is one of the most species‐rich families in Characiformes, with 145 valid species in 16 genera (Toledo‐Piza et al. 2024), occurring in all main basins of South America (Garavello and Britski 2003; Sidlauskas and Birindelli 2017). Two comprehensive studies, Winterbottom (1980) and Sidlauskas and Vari (2008), proposed phylogenetic hypotheses for anostomids based only on adult morphology, including mostly osteology. Despite these efforts, our understanding of the skeletal variation in anostomids is incomplete, especially regarding ontogenetic changes. Current data is limited to embryogenesis and early external larval stages of *Leporinus piau* Fowler 1941 (Borçato et al. 2004) and *Megaleporinus obtusidens* (Valenciennes 1837) (Sousa 2014; Perrotti et al. 2018), using histological techniques. Santos (1980; 1982) studied the external morphology of larvae of some Amazonian anostomids mainly interested in the development of colour patterns and snout shape. Nakatani et al. (2001) studied the development of the external morphology of eggs and larvae of six species of anostomids from the upper Paraná basin. They focused on providing data for species identification. Sanches et al. (2001) and Reynalte‐Tataje et al. (2001) provided data on the external morphology of *Leporinus friderici* (Bloch 1794) and *Megaleporinus macrocephalus* (Garavello and Britski 1988), respectively, with the later also being studied by Bonini‐Campos et al. (2019), discussing the species plastic responses to environmental changes. Ferreira (2015) described the external morphology of *Leporinus agassizii* Steindachner 1876 and focused on providing data for species farming. Machado‐Evangelista et al. (2015) studied the ecomorphology of juveniles and adults *Leporinus reticulatus* Britski and Garavello 1993 and showed distinct feeding habits during ontogenetic stages. Lastly, Santos et al. (2024) described the external morphology of *Rhytiodus microlepis* Kner 1858, providing data for larvae identification.

**Figure 1:**
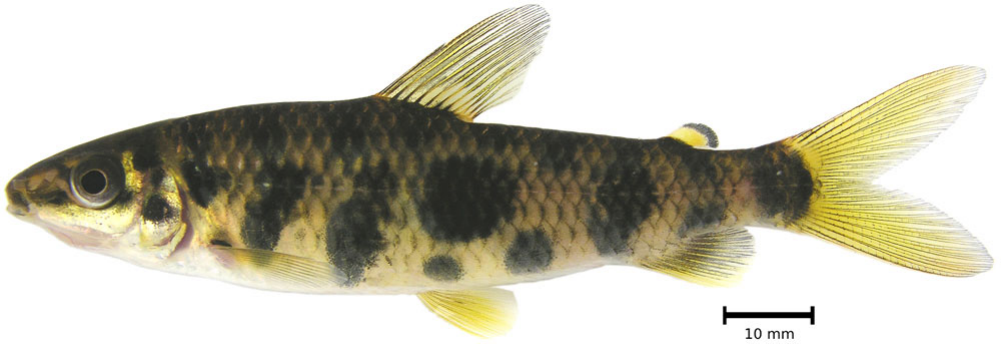
*Leporinus oliveirai*, uncatalogued live specimen. Photographed live by Mark Sabaj.

*Leporinus oliveirai* Ito et al. 2023 (Figure 1) is an anostomid recently described and endemic from the Braço Norte River, Tapajós basin, on the Serra do Cachimbo, in the state of Pará. The species gained some attraction in the ornamental fish trade nationally and internationally after being successfully raised in captivity by Piscicultura Tanganyika and introduced into the hobby (Lucanos 2021).

We obtained specimens from hatching to the juvenile state of *L. oliveirai*, allowing the description of the skeletal development. Our goal was to describe the ontogeny of the skeleton of *Leporinus oliveirai*, identify the sequence of formation and ossification of skeletal elements, and make comparisons with other Characiformes, and adult anostomids. The results might improve our understanding the development of characters, which helps understanding the group's evolution, by clarifying the homology and transformation series of some of the key morphological characteristics for the family, including those related to the maxilla and dentition (Vari 1983; Sidlauskas and Vari 2008; Sidlauskas et al. 2022).

## 2 Materials and Methods

Eighty‐nine specimens of *Leporinus oliveirai* Ito et al. (2023) were bred in captivity and deposited at Museu de Zoologia da Universidade Estadual de Londrina (MZUEL 20844, details about individuals are given in supplementary online material, Appendix 1). Reproduction was induced using Ovaprim in two doses for males (0.5 ml per kg of body weight) and one for females (0.4 ml per kg of body weight), with extrusion occurring within hours from the first hormone injection. Egg incubation occurred in trays at 27°C and specimens hatched approximately 55 h later. Fish larvae and juveniles from the day of hatching until sixty days post‐hatching were preserved in 4% calcium‐buffered formaldehyde. Additional adult specimens examined included MZUSP 119371 (2 cleared and stained (c&s), 103.6 and 104.1 mm SL). All specimens were transferred to 70% ethanol, and sixty‐three were cleared and stained (the specimens that were damaged during the examination of the external morphology were not c&s) following the method of Taylor and Van Dyke (1985), except for the duration of each step being much shorter. Cartilage terminology followed Cubbage and Mabee (1996), Bird and Mabee (2003) and Walter (2012). Bone terminology follows Weitzman (1962) with the modifications proposed by Sidlauskas and Vari (2008). Dissections were done according to the Ridewood method described in Bemis et al. (2004).

Morphological structures smaller than approximately 5–7 mm were photographed with a digital camera Olympus EP50 attached to a microscope Olympus CX43, and those larger than that were photographed with a digital camera DFC295 attached to a stereomicroscope Leica M205A. Notochord length (NL) was measured from the tip of the snout until the end of the notochord in specimens on yolk‐sac and pre‐flexion stages. Standard length (SL) was measured from the tip of the snout until the end of the hypural plate in specimens on flexion, post‐flexion and juvenile stages. Specimens were classified according to Nakatani et al. (2001), with juveniles being considered only specimens with full complement of the fin rays.

A table showing the development of each bony element was constructed considering the first presence of the element as cartilage to its complete ossification. Morphological description and ossification sequence were prepared considering body size, following previous studies that considered bone formation more related to body size than age (days post‐hatching, dph) (Bird and Mabee 2003; Mattox et al. 2014; Marinho 2022). All elements were organized by anatomical complexes. All skeletal elements were included in the table as separated elements, except for the following: infraorbitals 4 and 5; angular and articular; branchiostegal rays, gill rakers, fin rays and radials, supraneurals (with the exception of supraneural 3), all vertebra (except for centrum 1 to 4), ribs, neural and haemal arches (except for neural arches 3 and 4), neural and haemal spines, parapophyses and intermuscular bones.

## 3 Results

A total of 141 bony elements were identified and described in the skeleton of *Leporinus oliveirai* (Table 1).

**Table 1:** Developmental series for bony elements in *Leporinus oliveirai*, separated by anatomical complexes. The sequence of development of each bony element included cartilage precursors of chondral bones (blue) and the first sign of ossification (red). Lengths in mm NL/SL, with the larval period in white background ( < 27 mm SL), and juvenile period in green ( > 27 mm SL).

NL/SL mm ‐>	4.0–4.4	4.5–4.9	5.0–5.4	5.5–5.9	6.0–6.4	6.5–6.9	7.0–7.4	7.5–7.9	8.0–8.4	8.5–8.9	9.0–9.4	9.5–9.9	10.0–10.4	11.0–11.4	11.5–11.9	12.0–12.4	12.5–12.9	13.0–13.4	13.5–13.9	14.0–14.9	15.0–16.0	16.0–16.9	27.0–27.9	29.0–29.9	31.0–31.9	33.0–33.9
**Neurocranium ‐ Olfactory**																										
lateral ethmoid																										
mesethmoid																										
vomer																										
nasal																										
**Neurocranium ‐ Orbital**																										
parasphenoid																										
frontal																										
pterosphenoid																										
orbitosphenoid																										
**Neurocranium ‐ Otic**																										
sphenotic																										
prootic																										
pterotic																										
epiotic																										
parietal																										
**Neurocranium ‐ Occipital**																										
basioccipital																										
exoccipital																										
supraoccipital																										
**Infraorbital series**																										
antorbital																										
supraorbital																										
infraorbital 1																										
infraorbital 2																										
infraorbital 3																										
infraorbital 4 + 5																										
infraorbital 6																										
**Sclerotic bones**																										
sclerotic																										
**Jaws**																										
dentary																										
maxilla																										
premaxilla																										
anguloarticular																										
coronomeckelian																										
retroarticular																										
**Hyopalatine arch**																										
ectopterygoid																										
entopterygoid																										
quadrate																										
metapterygoid																										
hyomandibular																										
symplectic																										
autopalatine																										
**Opercular series**																										
opercle																										
interopercle																										
preopercle																										
subopercle																										
**Hyoid arch**																										
urohyal																										
branchiostegal rays																										
basihyal																										
interhyal																										
anterior ceratohyal																										
posterior ceratohyal																										
dorsal hypohyal																										
ventral hypohyal																										
**Branchial skeleton**																										
tooth plate PB4																										
tooth plate CB5																										
basibranchial 1																										
basibranchial 2																										
basibranchial 3																										
ceratobranchial 1																										
ceratobranchial 2																										
ceratobranchial 3																										
ceratobranchial 4																										
ceratobranchial 5																										
epibranchial 4																										
pharyngobranchial 4																										
hypobranchial 1																										
epibranchial 1																										
epibranchial 2																										
epibranchial 3																										
hypobranchial 2																										
hypobranchial 3																										
pharyngobranchial 1																										
pharyngobranchial 2																										
pharyngobranchial 3																										
gill rakers																										
**Weberian apparatus**																										
centrum 1																										
centrum 2																										
centrum 3																										
centrum 4																										
neural arch 3																										
neural arch 4																										
intercalarium																										
scaphium																										
os suspensorium																										
tripus																										
supraneural 3																										
claustrum																										
**Post‐Weberian axial skeleton**																										
vertebral centra																										
neural arches																										
haemal arches																										
ribs																										
haemal spines																										
parapophyses																										
neural spines																										
intermuscular bones																										
supraneurals																										
**Pectoral girdle**																										
cleithrum																										
supracleithrum																										
posttemporal																										
pectoral‐fin rays																										
postcleithrum 1																										
postcleithrum 2																										
postcleithrum 3																										
scapula																										
coracoid																										
mesocoracoid																										
extrascapular																										
**Pelvic girdle**																										
pelvic‐fin rays																										
basipterygium																										
**Dorsal fin**																										
dorsal‐fin rays																										
dorsal‐fin proximal radials																										
dorsal‐fin middle radials																										
dorsal‐fin distal radials																										
dorsal‐fin bony stay																										
**Anal fin**																										
anal‐fin rays																										
anal‐fin proximalradials																										
anal‐fin middle radials																										
anal‐fin distal radials																										
**Caudal fin**																										
principal rays																										
uroneural 1																										
uroneural 2																										
procurrent rays																										
hypural 1																										
hypural 2																										
hypural 3																										
parhypural																										
haemal arch PU2																										
haemal arch PU3																										
hypural 4																										
hypural 5																										
neural arch PU2																										
neural arch PU3																										
hypural 6																										
preural centra 2																										
preural centra 3																										
preural centra 1																										
ural centra 1																										
haemal spine PU2																										
haemal spine PU3																										
neural spine PU2																										
neural spine PU3																										
epural 1																										
epural 2																										
epural 3																										

### 3.1 Neurocranium: Olfactory Region (Figures 2, 3, 4)

**Figure 2:**
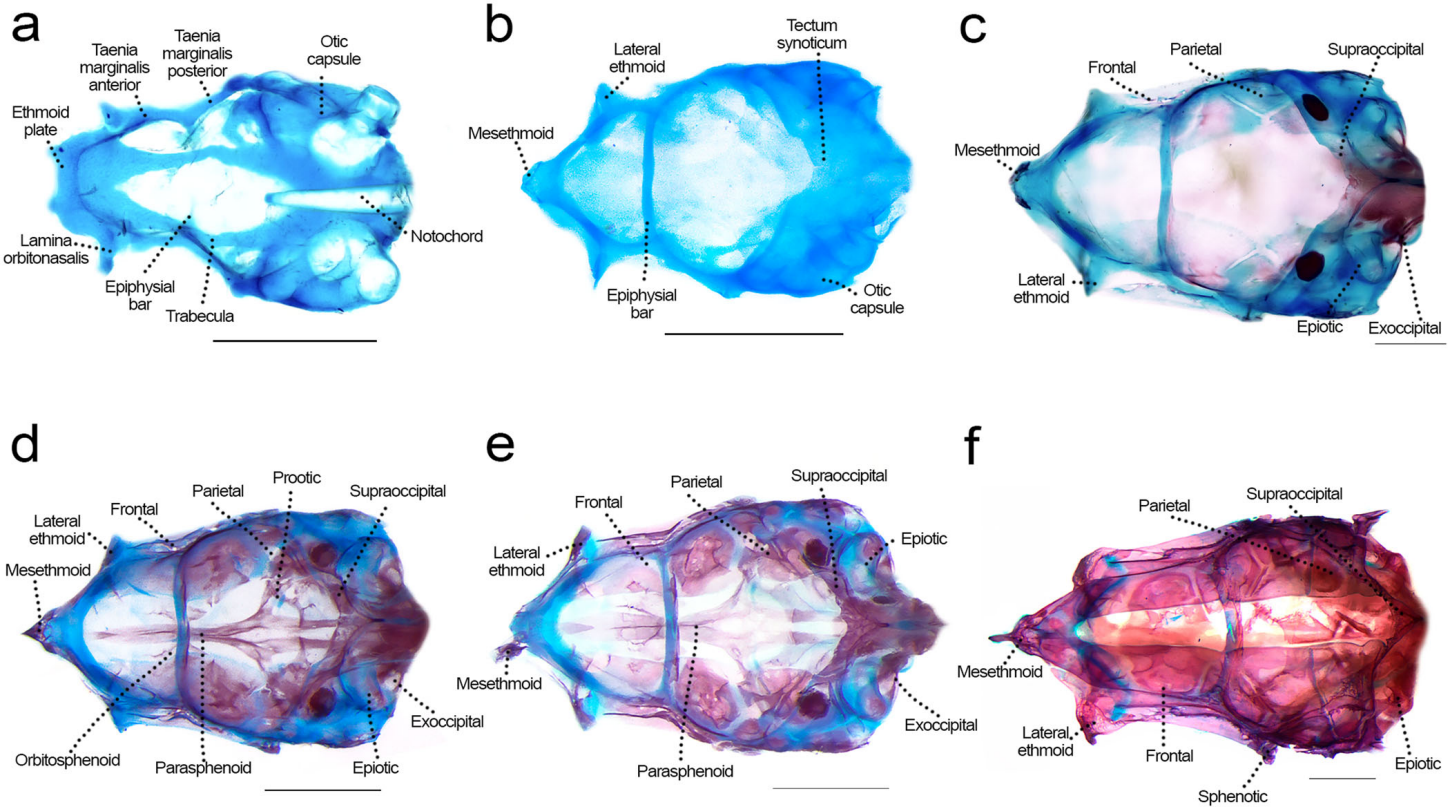
Neurocranium of *Leporinus oliveirai*, dorsal view, MZUEL 20844. (a) 6.0 mm NL. (b) 9.6 mm SL. (c) 11.8 mm SL. (d) 13.6 mm SL. (e) 14.5 mm SL. (f) 27.2 mm SL. Scale length: 1 mm.

**Figure 3:**
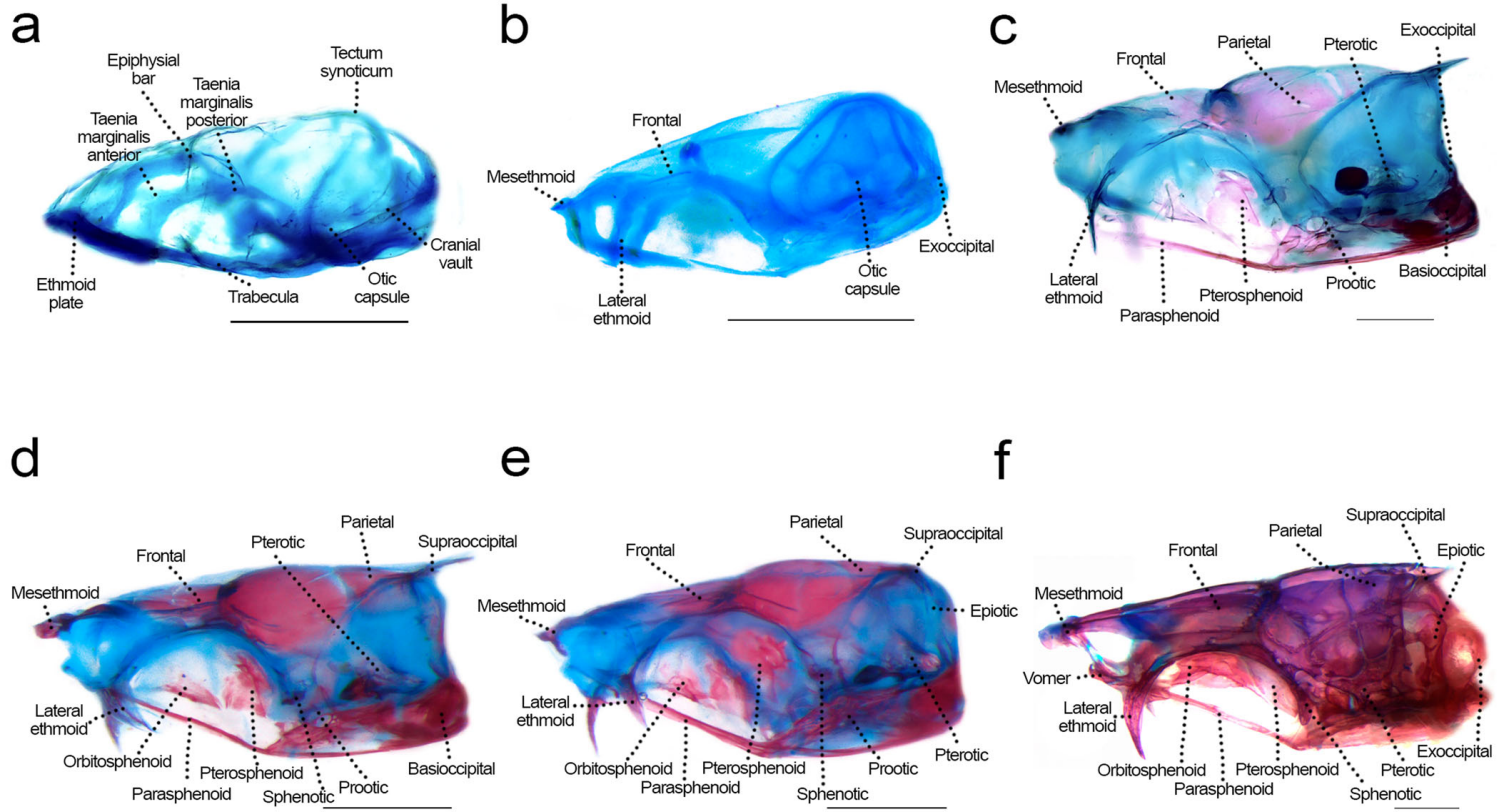
Neurocranium of *Leporinus oliveirai*, lateral view, MZUEL 20844. (a) 6.0 mm NL. (b) 9.6 mm SL. (c) 11.8 mm SL. (d) 13.6 mm SL. (e) 14.5 mm SL. (f) 27.2 mm SL. Scale length: 1 mm.

**Figure 4:**
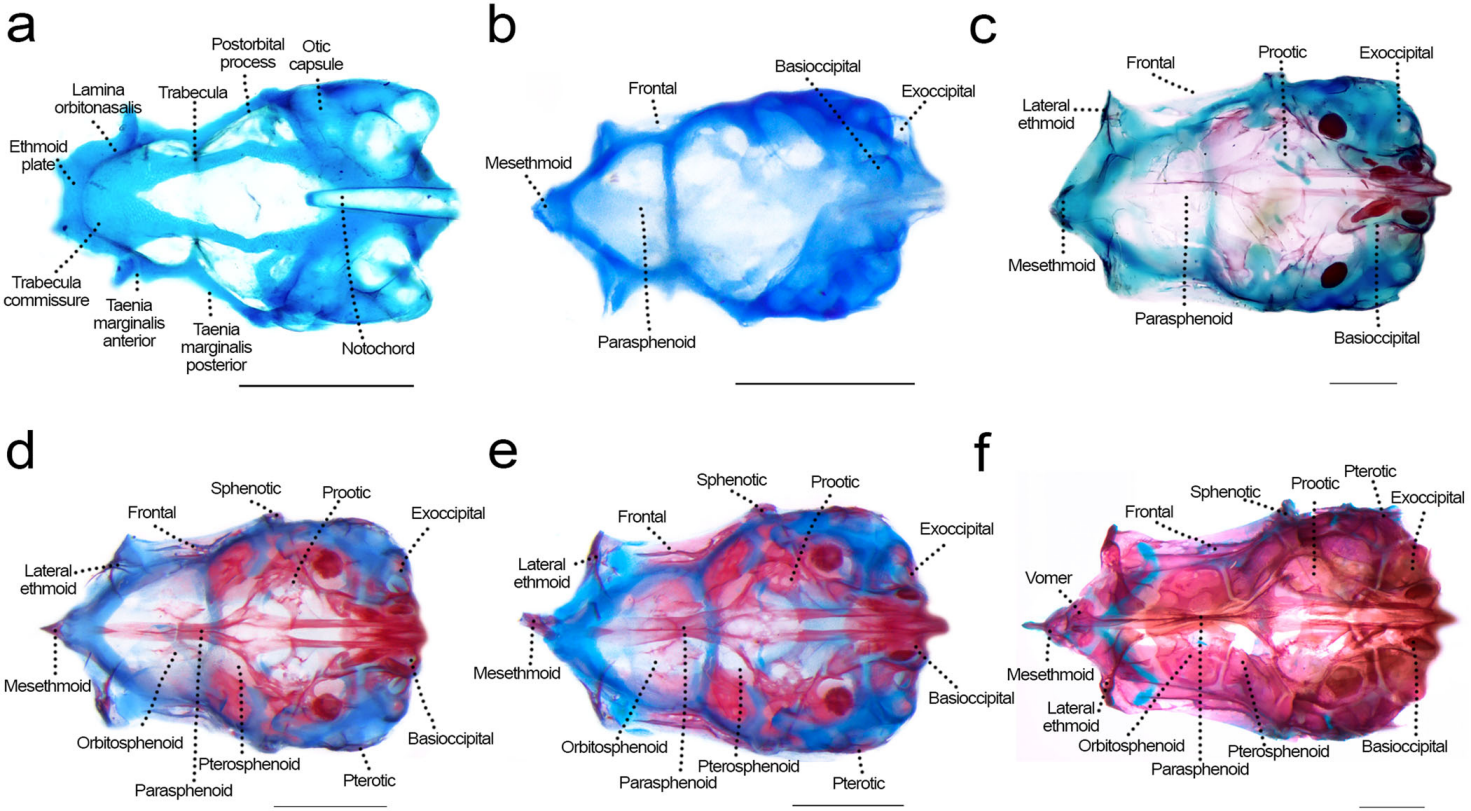
Neurocranium of *Leporinus oliveirai*, ventral view, MZUEL 20844. (a) 6.0 mm NL. (b) 9.6 mm SL. (c) 11.8 mm SL. (d) 13.6 mm SL. (e) 14.5 mm SL. (f) 27.2 mm SL. Scale length: 1 mm.

Sequence of ossification: (lateral ethmoid + mesethmoid) – vomer – nasal.

*Lateral ethmoid*: the lateral ethmoid is a paired chondral bone that starts to ossify at 9.6 mm SL on the medial side of the *lamina orbitonasalis*, expanding dorsally at 11.0 mm SL, and on the posterior margin at 12.1 mm SL, becoming more robust. At 13.6 mm SL the bone is almost completely ossified. The lateral ethmoid is a large triangular bone, curved posterolaterally, a condition seen also in adults.

*Mesethmoid*: the chondral ossification starts at 9.6 mm SL at the most anterior tip of the ethmoid plate, as a triangular shape resembling a boomerang, with a prominent tip anteriorly and the posterior base curved laterally. Ossification of the mesethmoid tip progresses at 11.7 mm SL, and at 12.1 mm SL starts to expand beyond the tip to the lateral margins in the posterior direction, and expands medially at 16.3 mm SL. The mesethmoid is completely ossified in juveniles (> 27.2 mm SL). In adults, it presents a distinct large and round anteroventral process.

*Vomer*: the vomer is a dermal ossification on the median line of the ventral surface of the ethmoid plate. It is one of the last bones of the neurocranium to develop, at 13.6 mm SL on its posterior portion, and later on its anterior portions where it spans laterally at 16.3 mm SL, finishing ossification in the juvenile stage (> 27.2 mm SL). The vomer is pentagonal with anterior projections that articulate with the autopalatine.

*Nasal*: the nasal appears at 14.5 mm SL as a thin splint of dermal bone, dorsal to the lateral ethmoid and lateral to the mesethmoid, the canal being half of its size. Ossification goes from posterior to anterior direction as the bone expands and becomes larger, becoming fully ossified at 27.2 mm SL, when it is in the same shape of the adult, i.e., larger at the base with a thinner tip posteriorly, the canal still located dorsally.

### 3.2 Neurocranium: Orbital Region (Figures 2, 3, 4)

Sequence of ossification: frontal – parasphenoid – pterosphenoid – orbitosphenoid (no trace of rhinosphenoid nor basisphenoid was observed).

*Parasphenoid*: the element is first seen at 9.6 mm SL as a paired splint ossification along the *trabecula*, united to the portion anterior of the epiphysial bar, and posteriorly reaching the basioccipital. At 11.0 mm SL, the ossification progresses on the middle and posterior portions, expanding more anteriorly at 11.8 mm SL, becoming completely ossified at 13.7 mm SL. The anterior portion is thin, with ascending processes reaching the prootic. Posteriorly, the parasphenoid bears two processes extending posteriorly to the basioccipital.

*Frontal*: the thin dermal bone appears at 9.6 mm SL, right above the orbit, lateral to the *taenia marginalis* cartilages and small, but joined at midline through the epiphyseal bar. At 11.8 mm SL the frontal expands to the medial margins, and both on anterior and posterior directions. At 15.7 mm SL the frontal is almost completely ossified except for the margins where it meets the mesethmoid anteriorly and the parietal posteriorly, giving shape to the anterior cranial fontanelle. On juveniles (27.2 mm SL) the bone is slightly larger and rectangular, like in adult individuals, with the anterior cranial fontanelle becoming smaller.

*Pterosphenoid*: the chondral ossification of the pterosphenoid starts at 11.7 mm SL, as a thin layer of bone ventral to the *taenia marginalis posterior*, expanding dorsally and posteriorly at 13.6 mm SL, and more to the anterior margin at 14.5 mm SL. The pterosphenoid is a robust and rectangular, almost completely ossified element by 15.7 mm SL.

*Orbitosphenoid*: the paired chondral ossification starts at 12.1 mm SL, on the ventral portion of the *taenia marginalis anterior*. Ossification progresses along with the development, expanding dorsally and anteriorly, until the bones become fully ossified at 27.2 mm SL. The orbitosphenoid is a large and rectangular paired bone, more square‐like on lateral view.

### 3.3 Neurocranium: Otic Region (Figures 2, 3, 4)

Sequence of ossification: sphenotic – prootic – (parietal + pterotic + epiotic).

*Sphenotic*: the chondral ossification starts at 9.6 mm SL, posterior to the orbit on lateral view. Ossification expands more in the posterior direction at 11.8 mm SL, and anterodorsally at 13.6 mm SL, until it is completely ossified at 16.3 mm SL, triangle‐shaped, bearing a pointed lateral spine that becomes more prominent in adults.

*Prootic*: the prootic first appears at 9.6 mm SL, between the parasphenoid and the sphenotic. Its ossification starts at 11.0 mm SL on the anterior and posterior margins, expanding more dorsally at 12.1 mm SL. At 15.7 mm SL the prootic is almost completely ossified, and still slightly translucid, somewhat triangular like the adult individuals.

*Parietal*: the parietal starts to ossify at 11.8 mm SL marginal to the fontanelle, posterior to the frontal, as a thin dermal bone. At 12.1 mm SL it expands on the medial side, and more to the anterior and posterior directions at 13.6 mm SL. At 16.3 mm the parietal is almost completely ossified, with some cartilage remaining at bone margins. In juveniles and adults the bone is large and rectangular.

*Pterotic*: the pterotic starts to ossify at 11.7 mm SL, with a single ossification site dorsal to the lateral margin of the otic capsule, with a projection that extends to the posterior direction. Ossification expands from the posterior to anterior margin, becoming fully ossified by 16.3 mm SL. The pterotic is somewhat rectangular, with a posterolateral process.

*Epiotic*: the chondral ossifications starts at 11.8 mm SL, both anteriorly and posteriorly, with the posterior margin expanding more to the anterior margin at 13.6 mm SL, and also medially and laterally. The epiotic is fully ossified in juveniles (27.2 mm SL), forming a three‐armed bridge.

### 3.4 Neurocranium: Occipital Region (Figures 2, 3, 4)

Sequence of ossification: basioccipital – exoccipital – supraoccipital.

*Basioccipital*: ossification starts at around 9.6 mm SL on the posterior margin, near the anterior tip of the notochord, and continues to expand anteriorly and posteriorly at 11.0 mm SL, forming a thin layer of chondral bone. At 11.7 mm SL ossification expands dorsally, and the bone keeps gradually developing until the juvenile stage when it is fully ossified (27.2 mm SL). In juveniles, the paired bones look like a butterfly, but in adult individuals the curved margins become a bit straighter, except for the posterior margin on lateral view which is still rounded. The basioccipital formes the entire occipital condyle, and the ventral portion of the foramen magnum.

*Exoccipital*: ossification starts at 11.0 mm SL on the posterior margin, quickly expanding anteriorly at the medial side at 13.6 mm SL, and more to the lateral margin at 14.5 mm SL. The exoccipital is almost fully ossified at 16.3 mm SL, in a rectangular shape like seen in adult specimens. The exoccipital formes the lateral and dorsal portions of the foramen magnum.

*Supraoccipital*: the supraoccipital starts to ossify at 11.7 mm SL on its anterior margin, immediately posterior to the parietal, expanding posteriorly and it is almost fully ossified at 14.5 mm SL, although still a bit translucid. At 16.3 mm SL the bone is fully ossified, small and triangular, becoming relatively larger in juveniles (> 27.2 mm SL) and adults.

### 3.5 Infraorbital Series (Figure 5)

**Figure 5:**
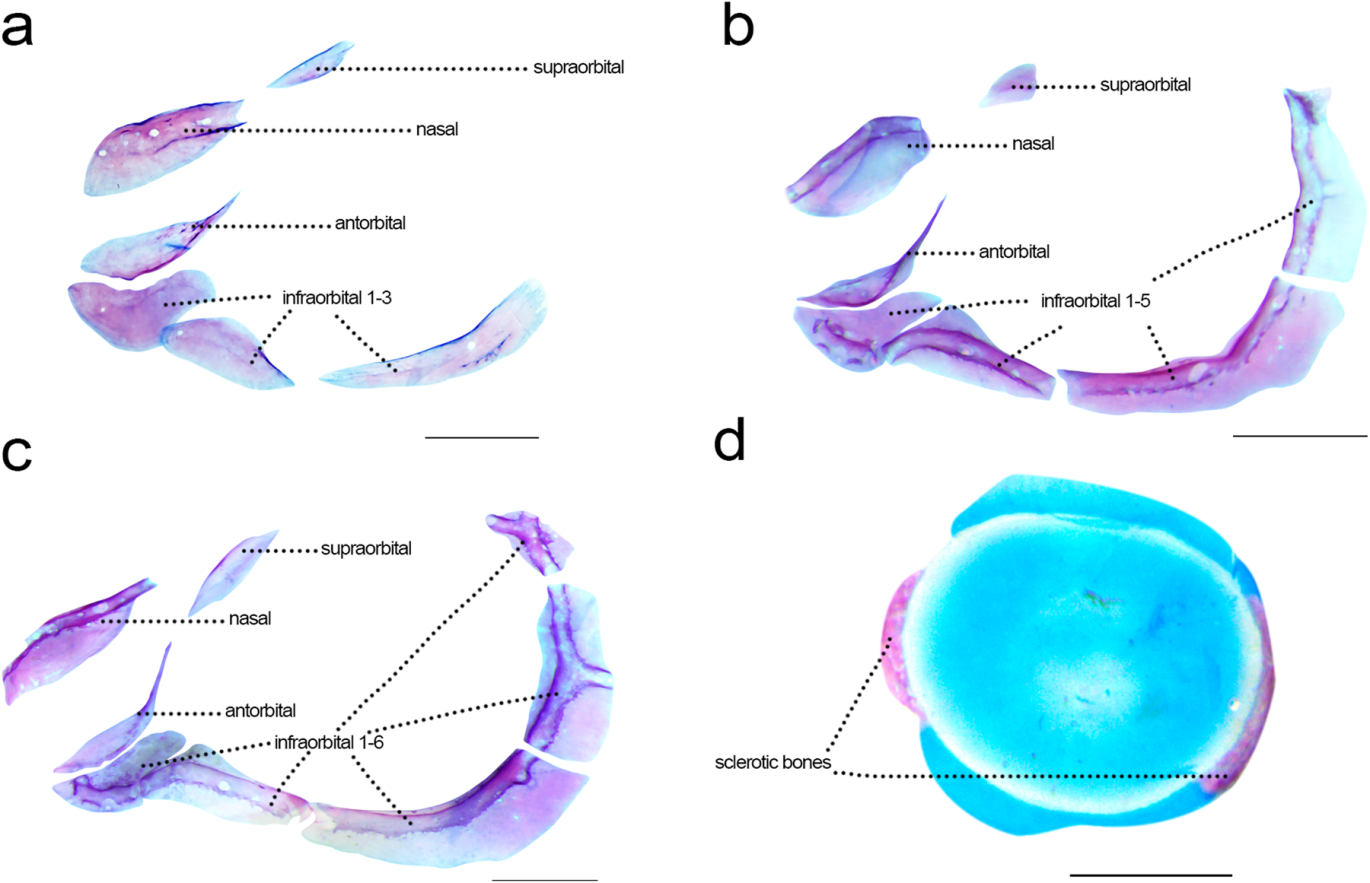
Infraorbital series and sclerotic bones of *Leporinus oliveirai*, MZUEL 20844, in lateral view. (a) 16.3 mm SL. (b) 27.2 mm SL. (c) 33.2 mm SL. (d) 27.2 mm SL. Scale length: 1 mm.

Sequence of ossification: (antorbital + supraorbital + infraorbital 1 + infraorbital 2) – infraorbital 3 – (infraorbital [4 + 5] + infraorbital 6 + sclerotic bones).

*Supraorbital:* the supraorbital can be first seen at 14.5 mm SL as a small oval thin dermal bone, above the orbit and posterodorsal to the lateral ethmoid. The supraorbital becomes more rectangular shaped at 15.7 mm SL, and more semicircular at 16.3 mm SL, with ossification finishing at 27.2 mm SL, when the bone is oval shaped like in adults, not bearing a canal.

*Antorbital*: first appears at 14.5 mm SL, anterior to the lateral ethmoid, large and oval shaped, with a thin posterior tip. Bony ossification starts posteroventral, with bone getting thinner during development. The antorbital is completely ossified at 27.2 mm SL, not bearing a canal.

*Infraorbitals*: The first infraorbital can be seen at 14.5 mm SL and beyond, immediately ventral to antorbital. Infraorbital 1 is larger anteriorly, and the posterior tip gets thinner during development, with a three‐pored canal. Infraorbital 2 is much more translucid and difficult to notice than the first at 14.5 mm SL, getting a more noticeable and triangular shape during development. Infraorbital 3 is distensible at 16.3 mm SL, as a thin long bone under the orbit. Infraorbital 6 appears at 27.2 mm SL with a tripartite canal, and ventral to it are infraorbital 4 and 5, developing as a single element, also with a tripartite canal, and this condition remains the same in the largest juvenile examined (33.2 mm SL).

*Sclerotic bones*: could only be seen in juvenile specimens (27.2‐33.2 mm SL), already ossified as two thin centers, respectively anterior and posterior to the orbit.

### 3.6 Jaws (Figure 6)

**Figure 6:**
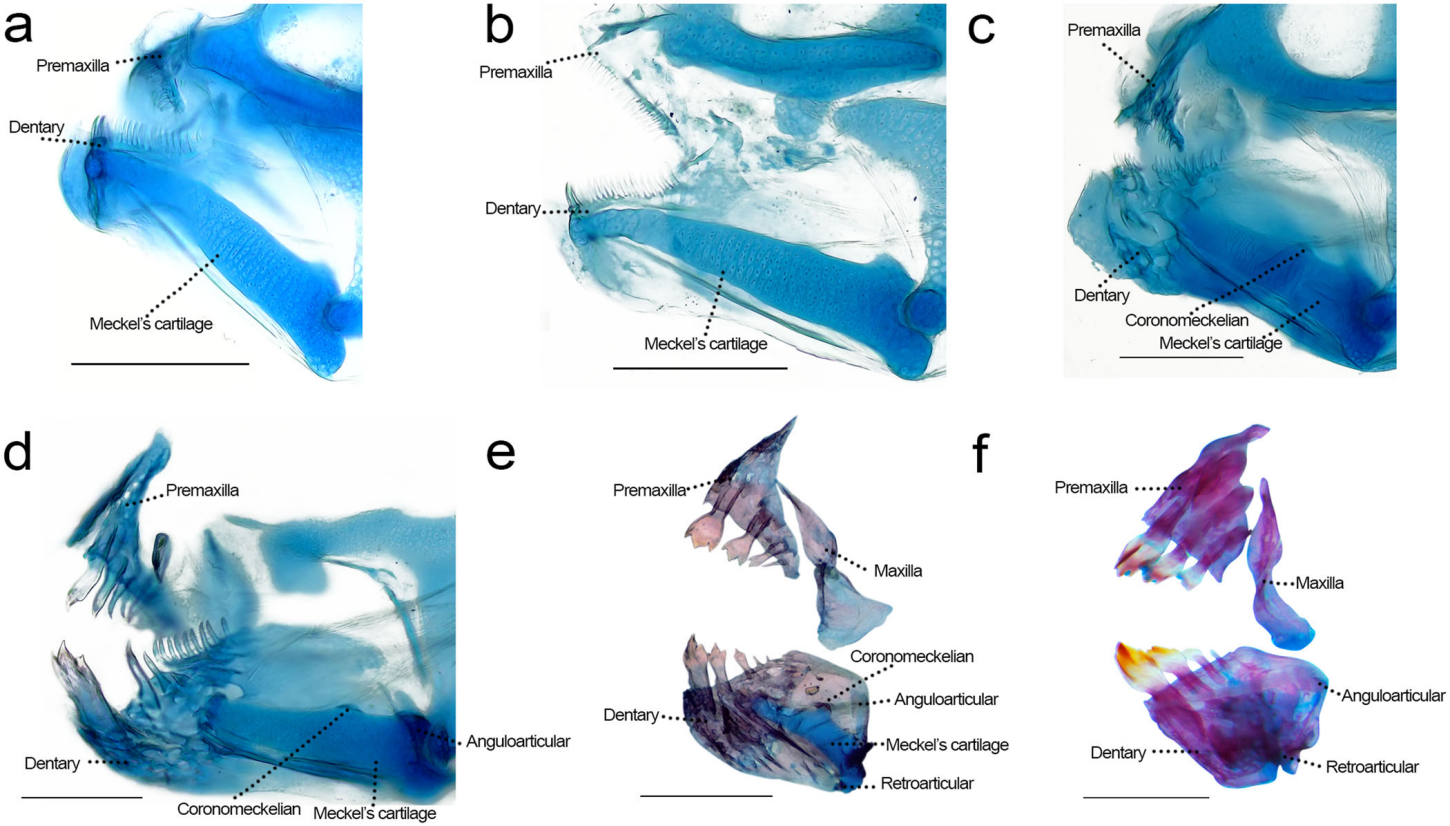
Upper and lower jaws of *Leporinus oliveirai*, MZUEL 20844, in medial view. (a) 6.4 mm NL. (b) 6.7 mm NL. (c) 8.4 mm SL. (d) 9.6 mm SL. (e) 11.8 mm SL. (f) 33.2 mm SL. Scale length: 1 mm.

Sequence of ossification: (dentary + premaxilla + retroarticular + coronomeckelian) – (maxilla + anguloarticular).

*Dentary*: the dentary can be first seen at 5.1 mm NL as a thin line of dermal bone at the dorsoanterior tip of Meckel's cartilage bearing one series of multiple translucid conical teeth. At 6.4 mm NL, the teeth remain translucid but have a more defined shape (around twenty teeth), and the anterior tip quickly expands in ventral direction. At 6.7 mm NL the dentary starts to expand posteriorly, with a small opening on the bone. The number of teeth apparently stays the same, although difficult to count as they are extremely translucid. At 8.4 mm SL, the ventral portion grows considerably, covering completely the anterior portion of the Meckel's cartilage, with around five small concavities along it. At the same time, on the dorsal side, a division can be seen at the anterior margin of the hole, with around six teeth staying on the posterior side of the bone, and the rest on the anterior portion. At 9.6 mm SL there are considerable changes of the teeth morphology and disposition, and the number of concavities in the dentary grows. The teeth on the front are replaced by around three of four tricuspid teeth, with nine small conical teeth that can be seen at the anterior portion of the dentary. By 11.0 mm SL the bone is almost completely ossified and similar in shape to the adult, sightly triangular shaped around the Meckel's cartilage, with three tricuspid teeth and the last one conical (some teeth were broken during dissection). The numerous concavities remain, but smaller, the most notable one being where the division of both portions was. At 12.1 mm SL the bone is completely ossified, and the teeth remain the same even as juveniles. The juveniles do not present notable concavities in the dentary like the larvae but it is still possible to pinpoint where the division was.

*Maxilla*: it can be first seen at 5.1 mm NL as a small thin line of bone, half of the size of the premaxilla and posterodorsal to it. The presence of the maxilla is hard to pinpoint at the early stages as it is not articulating with the jaws, being a bit far away laterally and hanging by the thin remains of skin in the mouth, which may have been lost in a few specimens during the procedures. The bone enlarges following the development of the rest of the jaws (6.5 mm NL) and the position becomes vertical to the premaxilla (still posterior to it; 6.8 mm NL). By 8.4 mm SL the bone becomes hourglass shaped. At 9.6 mm SL the maxilla is larger except for the middle, extending from the middle portion of the premaxilla to the dorsal margin of Meckel's cartilage. By 11.0 mm SL, the bone start to get stained with alizarin red and becomes completely stained at around 13.7 mm SL; at this stage the maxilla is still hourglass shaped except the dorsal tip being thin and elongate and, at 16.3 mm SL, the maxilla is similar to that of the adults. The opening where the primordial ligament goes through is possible present since the bone acquires its hourglass shape (8.4 mm SL), in the middle portion, but the bone is still thin and fragile in this state, which makes visualization difficult. The opening becomes more prominent at 11.0 mm SL, and two or three small concavities can also be seen. The maxilla is toothless throughout the development.

*Premaxilla*: can be first seen at 5.1 mm NL as a thin line of dermal bone anterior to the ethmoid plate with one series of numerous conical teeth on its ventral margin. At 6.4 mm NL the premaxilla has a more distinct triangular shape and bears around fifteen conical teeth, all small and translucid, and it expands posteroventral at 6.7 mm NL. At 9.0 mm SL the premaxilla gets more triangular shaped, being restricted to the anterior portion of the ethmoid plate. At 9.6 mm SL the first two teeth are being replaced by tricuspid teeth and only around five conical teeth staying in the rest of the series. By 11.0 mm SL ossification is almost complete and the shape of the premaxilla and teeth morphology is almost identical to the adult, except for the presence of a fourth (and in a few specimens, a fifth) conical teeth. The fourth teeth on the premaxilla only disappears on the juveniles (27.2–33.2 mm SL). Just like the dentary, a few small concavities can be seen in the entire bone during development, by 12.2 mm SL those are not visible anymore.

*Anguloarticular*: the anguloarticular starts to develop at 5.2 mm NL as a small thin line at the posterodorsal margin of Meckel's cartilage, articulating with the palatoquadrate cartilage, and also on the ventral margin of the posterior tip of Meckel's cartilage. At 9.6 mm SL, this element forms a rectangle, covering the entire posterior margin of the cartilage. Ossification starts at 11.0 mm SL and finishes at 11.7 mm SL.

*Retroarticular*: first visualized at 6.4 mm NL as tiny thin line on posteroventral portion of Meckel's cartilage, becoming distinctly developed at 9.6 mm SL. At 11.0 mm SL it is a small triangular bone, completely ossified, growing a little as the larvae develop, becoming more square‐like.

*Coronomeckelian*: first seen at 8.3 mm SL, translucid, posterior to the middle portion of the dorsal margin of the Meckel's cartilage. Ossification starts at 9.6 mm SL and quickly finishes, the coronomeckelian grows slowly to a semicircular shape bone and, in juveniles the bone is small and hardly distinguishable.

### 3.7 Hyopalatine Arch (Figure 7)

**Figure 7:**
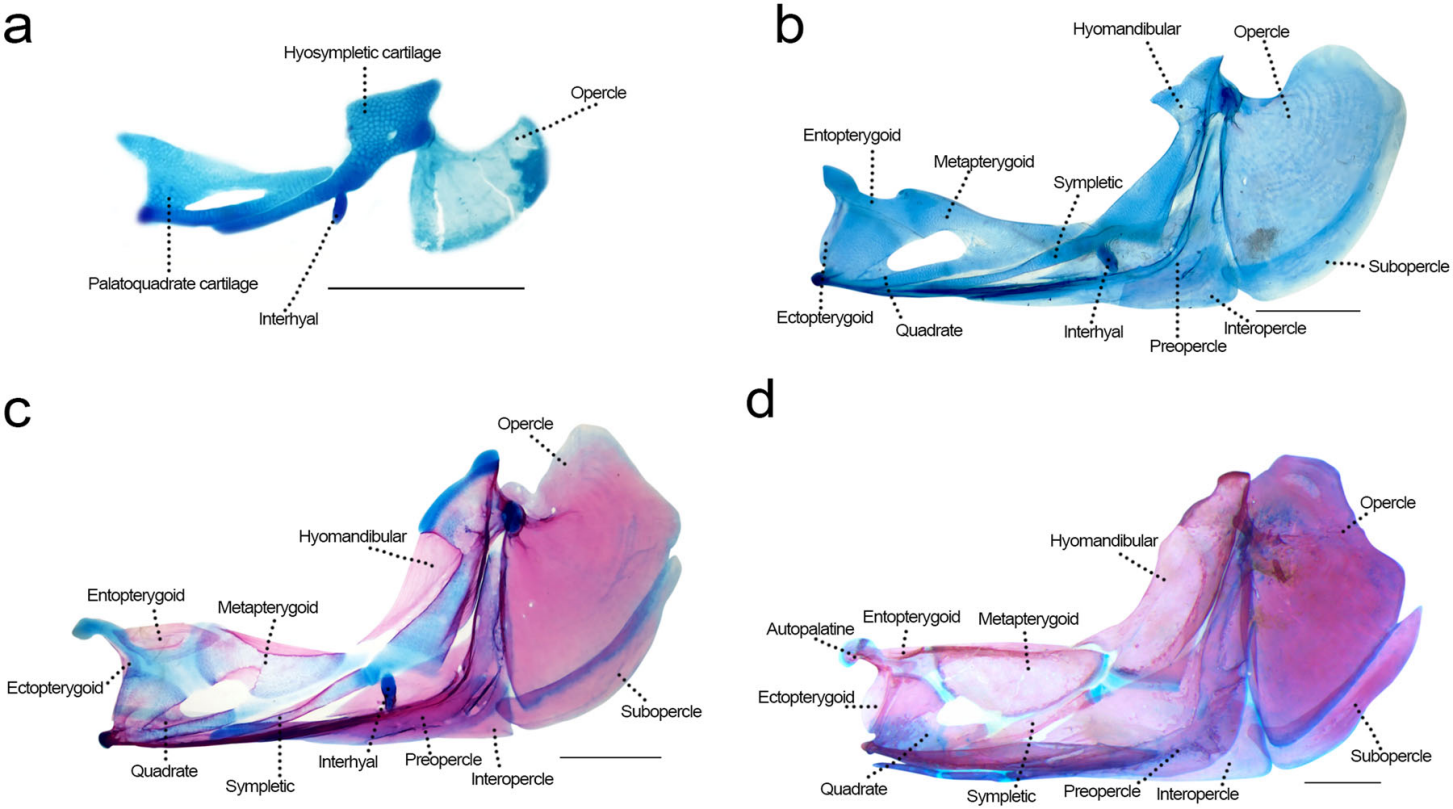
Suspensorium of *Leporinus oliveirai*, MZUEL 20844, in lateral view. (a) 6.8 mm NL. (b) 10.9 mm SL. (c) 12.1 mm SL. (d) 33.2 mm SL. Scale length: 1 mm.

Common sequence of ossification: (quadrate + hyomandibular + sympletic) – (ectopterygoid + metapterygoid) – entopterygoid – autopalatine.

*Quadrate*: the chondral ossification starts at 11.0 mm SL, around the ventral tip of palatoquadrate cartilage, ventral to the metapterygoid‐quadrate fenestra, and expands anteriorly at 11.7 mm SL and posteriorly at 12.1 mm SL. At 14.5 mm SL the quadrate is almost completely ossified, except for a tiny portion of cartilage anteroventrally. The quadrate is expanded posterolaterally covering part of the preopercle, sympletic and hyomandibular.

*Hyomandibular*: ossification starts at the posterior portion of the hyosymplectic cartilage at 11.0 mm SL, and anteriorly at 11.7 mm SL as a bony lamina that develops ventrally. At 14.5 mm SL ossification is almost completely ossified, except for the cartilage that connects it to the neurocranium and its ventralmost portion near the symplectic. The hyomandibular bears a triangular process on the anteroventral margin, and the processus opercularis somewhat triangle and directly posteriorly on the posterodorsal margin.

*Symplectic*: ossification starts at 11.0 mm SL at the middle portion of the cartilage ventral to the quadrate cartilage, and ventrally at 11.7 mm SL, expanding to both sides and acquiring a more precise rod‐like shape. Ossification finishes at 14.5 mm SL, except for the tips on both sides, where it articulates posteriorly with the hyomandibular and anteriorly with the quadrate.

*Ectopterygoid*: the bone starts to ossify at 11.0 mm SL, vertical relative to the body axis, becoming fully ossified by 11.8 mm SL, with the dorsal tip curving following the autopalatine cartilage. The ectopterygoid is a sightly oval bone, in the adults the ventral portion is a bit larger and rounded.

*Metapterygoid*: the chondral ossification starts at 11.0 mm SL at the dorsal margin, and ventrally at 11.7 mm SL, expanding anteriorly and posteriorly, involving its cartilage precursor. The ossification is complete at 14.5 mm SL, with remains of cartilage connecting it to the entopterygoid and ectopterygoid anteriorly, and to the quadrate and hyomandibular ventrally. The bone is triangular with rounded margins.

*Entopterygoid*: the entopterygoid is a small bone that starts to ossify at 11.7 mm SL, dorsal to the palatoquadrate cartilage, quickly encircling the cartilage and expanding ventrally at 12.1 mm SL, being completely ossified by 13.6 mm SL, when it is more rounded, similar to that of adults.

*Autopalatine*: the autopalatine cartilage can be seen as early as 5.1 mm NL, as an extension of the palatoquadrate cartilage. During development this palatine process acquires curvature, like the condition present in adults, and starts to ossify at 11.8 mm SL at the anterior tip. The autopalatine could only be seen fully ossified in juveniles (27.2 mm SL). A small oval autogenous cartilage can be seen during the entire development at the dorsoanterior tip of the autopalatine, possibly being its connection to the ethmoid plate and consequently the neurocranium. This small piece of cartilage remains in adulthood.

### 3.8 Opercular Series (Figure 7)

Sequence of ossification: (opercle + interopercle + preopercle) – subopercle.

*Opercle*: the opercle is a dermal bone that starts to develop at 7.3 mm NL, staining with alizarin at 9.6 mm SL on its anterior and dorsal margins, expanding posteriorly to the middle of the cartilage, and being fully ossified at around 13.6 mm SL. The opercle is the largest bone in the opercular series, somewhat triangular with rounded corners, overlapping the subopercle.

*Interopercle*: ossification starts at the middle portion of the dorsal margin at 9.6 mm SL, expanding ventrally both anteriorly and posteriorly at 11.8 mm SL, ossification finishing at 13.6 mm SL. The bone is mostly overlapped by the preopercle, and overlaps the anterior tip of subopercle.

*Preopercle*: the ossification starts at 9.6 mm SL at the anterodorsal margin, expanding and circling the bone at 11.8 mm SL, until its fully ossified by 13.6 mm SL. The preopercle is overlapped anteriorly by the lateral projection of the quadrate, and the posterior portion is curved in the dorsal direction, overlapping the interopercle. Preopercular sensory canal with one pore directed posteriorly on the ventral portion of the bone, and another directed ventrally, and the posteroventral corner of the bone.

*Subopercle*: can be first seen at 8.4 mm SL, and the subopercle starts to stain with alizarin at the small anterior margin at 11.0 mm SL, slowly expanding to the posterior margin until its fully ossified at 13.6 mm SL. A distensible bone overlapped on the anterior tip by the interopercle, and all of its dorsal margin by the opercle.

### 3.9 Hyoid Arch (Figure 8)

**Figure 8:**
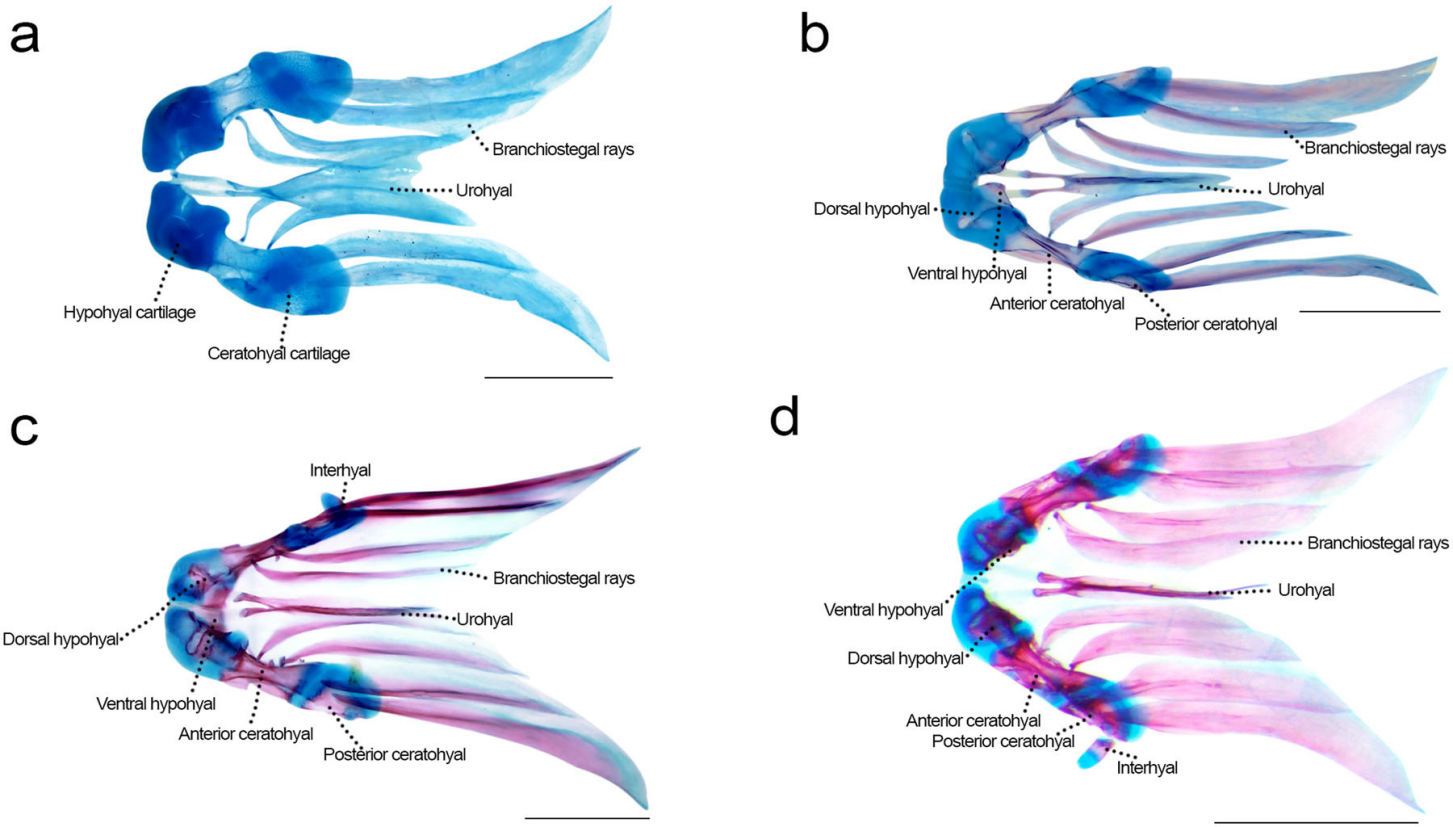
Hyoid arch of *Leporinus oliveirai*, MZUEL 20844, in dorsal view. (a) 11.0 mm SL. (b) 11.7 mm SL. (c) 11.8 mm SL. (d) 14.5 mm SL. Scale length: 1 mm.

Sequence of ossification: branchiostegal rays – (urohyal + interhyal + basihyal + ceratohyal + hypohyal).

*Branchiostegal rays*: the eight branchiostegal rays can be first seen at 6.4 mm NL as thin lines connected to the ceratohyal cartilage, expanding posteriorly and laterally, reaching a spatula‐shape much similar to that in adults at 8.7 mm SL. Ossification starts at 11.0 mm SL, expanding dorsoventrally, becoming fully ossified at 14.5 mm SL.

*Urohyal*: a dermal bone that appears at 6.4 mm NL as a thin line that develops at 8.7 mm SL, connected to the ventral hypohyal by the anterior bifurcation where ossification starts at 11.7 mm SL and expands to the ventral direction, finishing at 12.1 mm SL. The urohyal is a somewhat triangular bone, with a concave base.

*Ceratohyal*: both the anterior and posterior portions start to ossify chondraly simultaneously at 11.7 mm SL, on the middle portion and posterodorsal margin, respectively, of the ceratohyal cartilage, finishing at 13.6 mm SL, only remaining cartilage where both portions articulate and where the anterior ceratohyal articulates to the dorsal hypohyal.

*Hypohyal*: the dorsal and ventral portions of the hypohyal develop simultaneously, starting to ossify at 11.7 mm SL anteroventrally to the ceratohyal cartilage, expanding dorsally at 13.6 mm SL and finishing at 15.7 mm SL.

*Basihyal*: ossification starts at 11.7 mm SL at the posterior margin of the basihyal cartilage and expands to the anterior margin, only ventrally at the anteriormost tip, leaving the centre of the tip cartilaginous at around 14.5 mm SL, and in adults.

*Interhyal*: ossification starts at 11.8 mm SL on the margin that articulates with the ceratohyal cartilage, and its fully ossified at 12.1 mm SL, except for the dorsal tip that articulates with the suspensorium, that remains cartilaginous, as in adults.

### 3.10 Branchial Skeleton (Figure 9)

**Figure 9:**
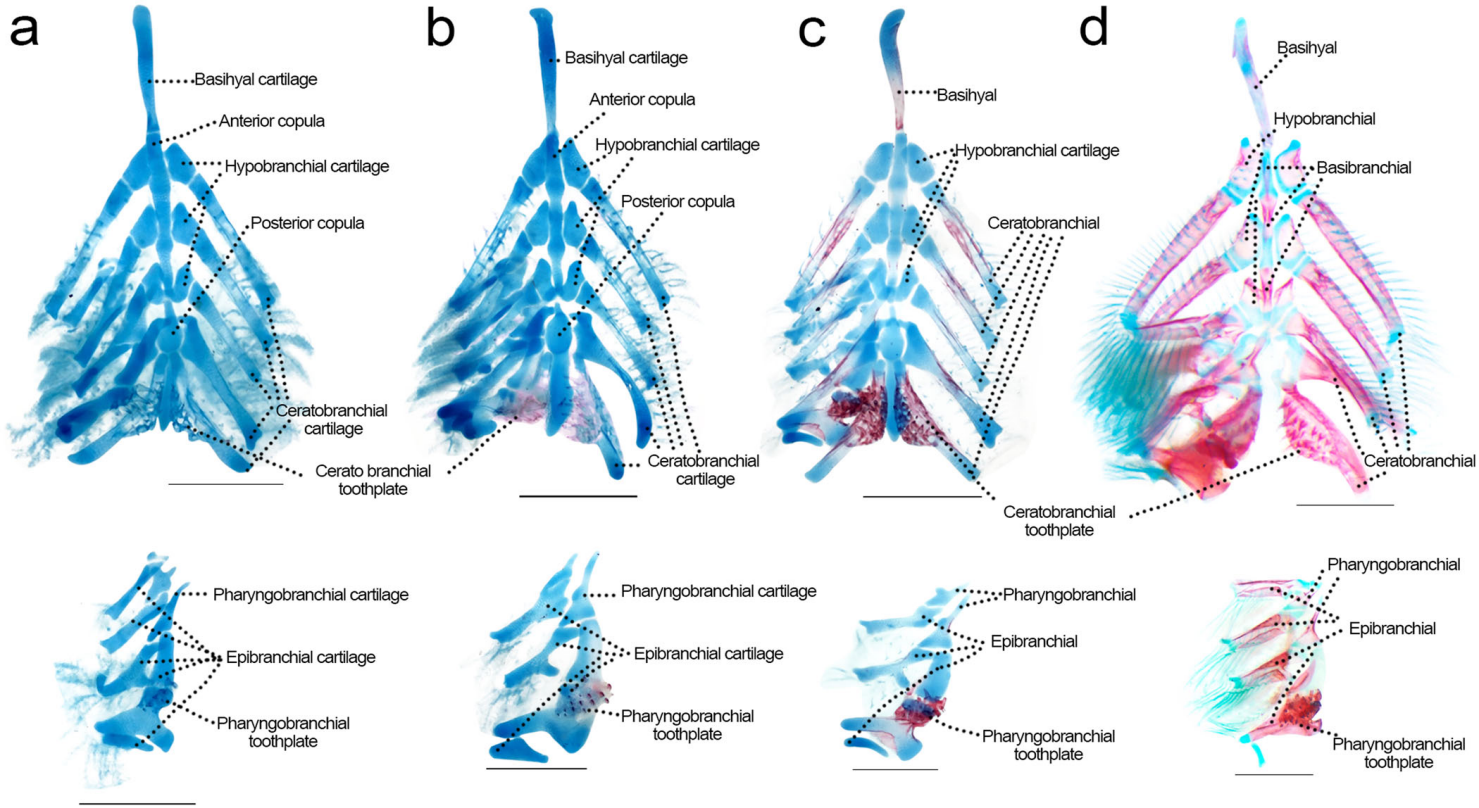
Branchial apparatus of *Leporinus oliveirai*, MZUEL 20844, in dorsal view (above), and dorsal elements of the right side in ventral view (below). (a) 9.8 mm SL. (b) 11.0 mm SL. (c) 12.2 mm SL. (d) 27.2 mm SL. Scale length: 1 mm.

Sequence of ossification: tooth plates – (ceratobranchials 1‐5 + basibranchials 1‐3) – (epibranchial 4 + pharyngobranchial 4) – (hypobranchial 1 + epibranchial 1‐3) – hypobranchial 2‐3 – pharyngobranchial 1‐3 – gill rakers.

*Pharyngeal jaws*: the pharyngeal jaw is formed by the tooth plates appearing at 9.6 mm SL, it quickly begins ossification and bearing teeth, becoming completely ossified at 11.8 mm SL.

*Ceratobranchials*: the chondral ossification starts at 11.8 mm SL on the middle portion of the cartilage, from the lateral margins to the centre, encircling the cartilage and expanding in both directions until it is fully ossified at 27.2 mm SL.

*Hypobranchials*: the first hypobranchial starts to ossify at 14.5 mm on the anterior margin, with the other two hypobranchials starting at 15.7 mm SL. In the juveniles (27.2 mm SL), the margins that connect to the ceratobranchials and basibranchials still encompass more cartilage than in adults.

*Basibranchials*: ossification starts at 11.8 mm SL simultaneously on the middle portion of the three basibranchials, and encircling the cartilage and expanding to both ends, with tips still cartilaginous in adults.

*Epibranchials*: ossification starts on the fourth epibranchial at 12.1 mm SL, and the other three at 14.5 mm SL and the ossification is completed in juveniles (27.2 mm SL).

*Pharyngobranchials*: ossification of the fourth pharyngobranchial starts at 12.2 mm SL, and the other three at 15.7 mm SL, finishing only in juveniles (27.2 mm SL).

*Gill rakers*: the first gill rakers can be seen at around 9.5 mm SL, associated with ceratobranchials 1 and 2, five on each. By 10.1 mm SL each ceratobranchial has five rakers, and the epibranchials present around two rakers each. The gill rakers grow in numbers through development. All epibranchials support around five rakers on the juvenile stage while the ceratobranchials support two series of multiple rakers.

### 3.11 Weberian Apparatus and Associated Centra (Figure 10)

**Figure 10:**
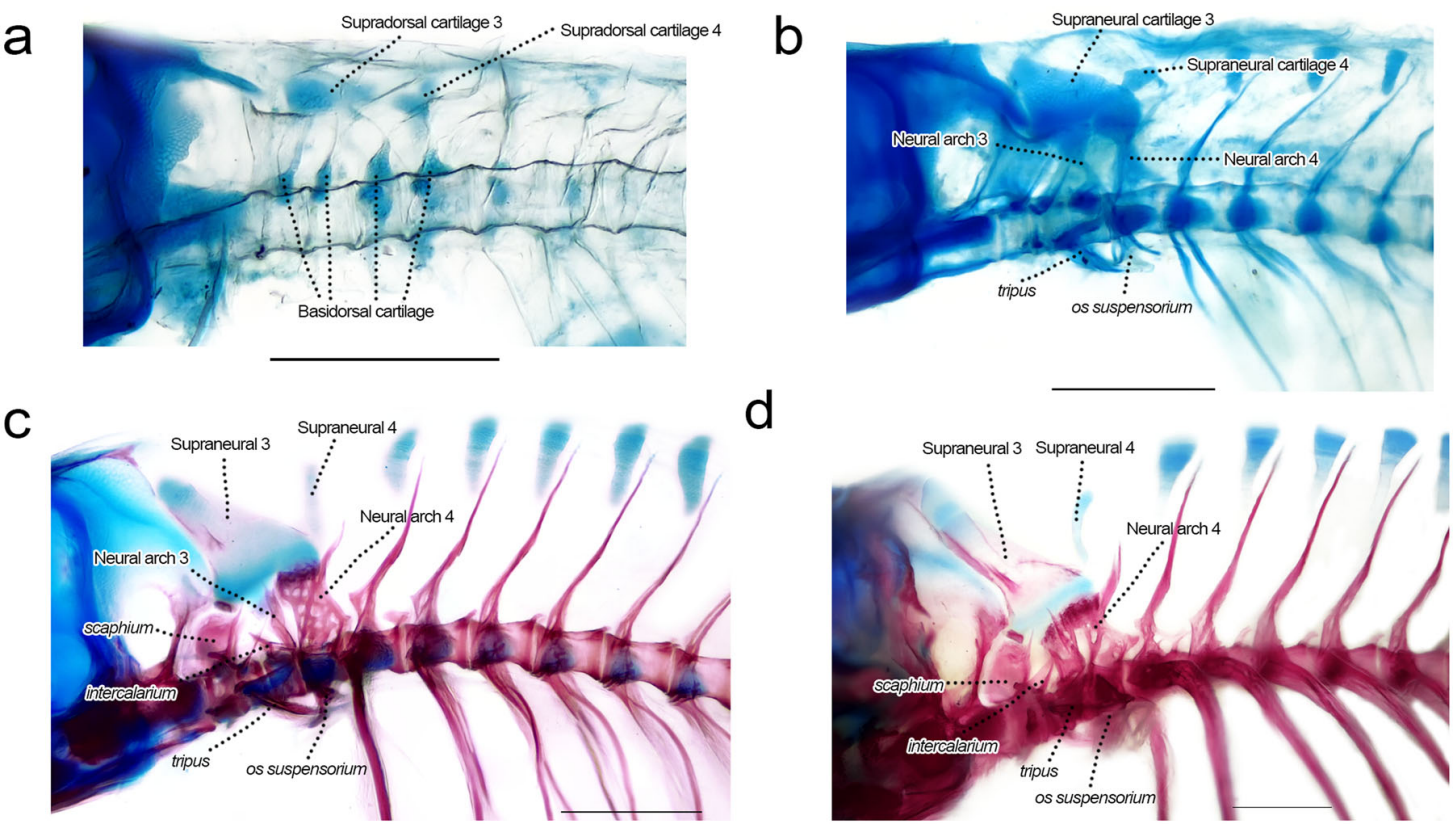
Weberian apparatus of *Leporinus oliveirai*, MZUEL 20844, in lateral view. (a) 7.9 mm SL. (b) 9.7 mm SL. (c) 12.2 mm SL. (d) 16.3 mm SL. Scale length: 1 mm.

Sequence of ossification: (centrum 1–4 + neural arch 3–4) – (*intercalarium* + *scalphium)* – (*os suspensorium* + *tripus* + supraneural 3 + *claustrum)*.

*Centra 1–4*: the first four vertebral centra start to ossify at 9.6 mm SL at the anterior and posterior margins of each vertebra, encircling the notochord, becoming fully ossified at 11.7 mm SL. The first three centra are smaller than the fourth, which supports the large neural arch 4. Centrum 2 presents a lateral process, elongated and directed anteriorly. Centrum 3 also presents a lateral process, smaller and thinner, located dorsally and also directed anteriorly, and this process is almost indistinguishable in lateral view, being more easily spotted on dorsal and ventral views.

*Neural arches 3 and 4*: the neural arches 3 and 4 start to develop at 9.6 mm SL around the basidorsal cartilages, expanding dorsally, quickly ossifying. At 11.7 mm SL the neural arch 4 is hourglass shaped with a sharp posterodorsal spine. At 12.2 mm SL it becomes more rectangular, and neural arch 3 is also larger and closer to neural arch 4. In juveniles (27.2 mm SL) both are barely distinguishable, ventral to the well‐developed supraneural 3.

*Intercalarium*: the *intercalarium* is a chondral bone anterior to the neural arch 3, a thin line of bone showing at 11.0 mm SL and already ossified at 11.7 mm SL, with dorsal portion bending posteriorly with development.

*Scaphium*: a small thin bone anterior to the *intercalarium*, inclined posteriorly, also starting to ossify at 11.0 mm SL and quickly finishing, presenting at 11.7 mm SL a semicircular anterior process.

*Os suspensorium*: the *os suspensorium* starts to develop at around 9.8 mm SL on the ventrolateral margins of the fourth centrum, starting to ossify at 11.7 mm SL, almost fully ossified at 12.2 mm SL except for a small portion on the base. The bone remains small during development and directed slightly anteriorly, ventral to the *tripus*.

*Tripus*: starts to ossify at 11.7 mm SL, around the somewhat triangular basiventral cartilage lateral to centrum 3, finishing at 13.6 mm SL, when the *tripus* is in a similar position and shape to that of juveniles and adults.

*Supraneural 3*: ossification starts at 11.7 mm SL on the anterior margin of the dorsal portion, expanding laterally to the ventral margin, and at 12.2 mm SL on the posterior margin, encircling the cartilage in both directions. Supraneural 3 was only seen fully ossified in juveniles, at 27.2 mm SL.

*Claustrum*: the *claustrum* is a small and thin bone first seen at 11.8 mm SL, already mostly ossified, on the lateral posterodorsal margin of the *scaphium*.

### 3.12 Post‐Weberian Axial Skeleton (Figure 11)

**Figure 11:**
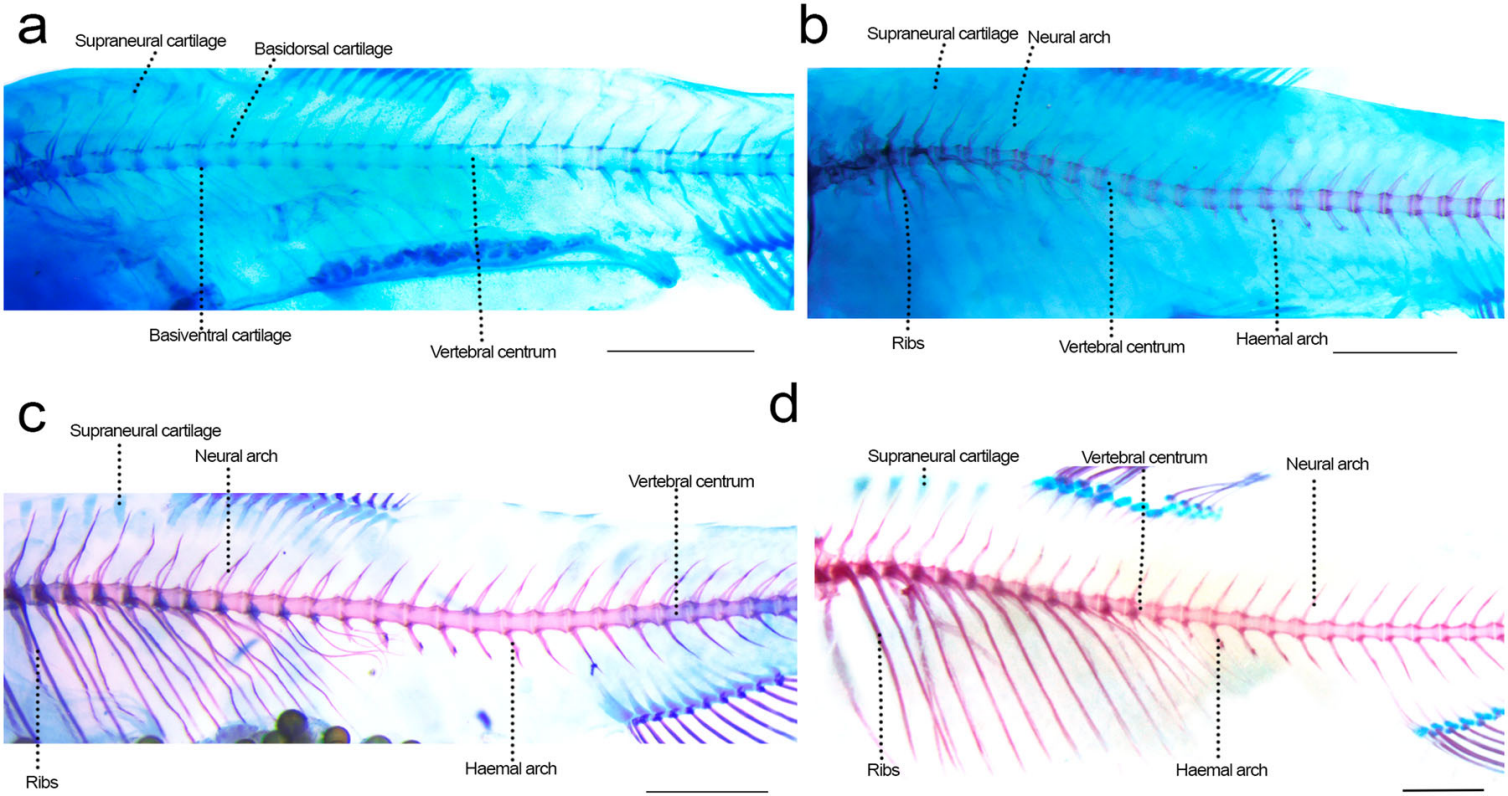
Axial skeleton of *Leporinus oliveirai*, MZUEL 20844, in lateral view. (a) 10.0 mm SL. (b) 11.7 mm SL. (c) 12.2 mm SL. (d) 16.3 mm SL. Scale length: 1 mm.

Sequence of ossification: (vertebral centra + neural and haemal arches + ribs) – (haemal spines + parapophyses) – (neural spines + intermuscular bones) – supraneurals.

*Vertebral centra*: the post‐Weberian vertebral centra start to develop at 6.2 mm NL around the notochord, with a bidirectional chondral ossification following at 9.6 mm SL. The anterior and posterior margins start to ossify and encircle the cartilage of each centra and it progresses quickly, with the middle portion of the notochord being the last to fully form. All centra are formed at 12.1 mm SL. Zygapophyses start to develop first at the posterior portion of the vertebrae at 11.6 mm SL, reaching the anterior portion at 13.5 mm SL, becoming just sightly more robust during development.

*Neural arches and spines*: the neural arches start to ossify at 9.8 mm SL, where it is connected to the vertebral centra, and expands on dorsal direction as development progresses, becoming complete ossified at 14.5 mm SL. The neural spines start to develop at 12.1 mm SL, on all vertebrae around the same time and ossification finishes quickly (13.2 mm SL).

*Haemal arches and spines*: the haemal arches start to ossify at the same time as the neural arches (9.8 mm SL), at the base where it is connected to the vertebrae, quickly expanding ventrally and finishing ossifying at 12.1 mm SL. The haemal spines start to develop at 11.0 mm SL and completely ossify shortly after at 11.8 mm SL.

*Ribs*: the anteriormost ribs start to ossify at 9.8 mm SL, with the next ribs following short after, with the posteriormost ribs finishing development at around 16.3 mm SL.

*Supraneurals*: five supraneurals are present anterior to the dorsal‐fin pterygiophores; their chondral ossification starts at 14.5 mm SL at both anterior and posterior margins of the cartilage, and the full ossification was only seen in juveniles (27.2 mm SL).

*Intermuscular bones*: the intermuscular bones start to develop at 12.1 mm SL with the epineurals, at the posterior portion of the body, with the epipleurals appearing almost simultaneously at 12.3 mm SL. While they appear already ossified, the bones are barely visible at first, becoming a bit more prominent at 13.7 mm SL and developing in the anterior direction. Juveniles present epineurals on all post‐Weberian vertebrae and epipleurals on the caudal region.

*Parapophyses*: the parapophysis of all vertebrae starts to develop at around 11.0 mm SL, chondral ossifying around the basiventral cartilage, finishing at around 13.5 mm SL. Their development follows the expansion of the ribs.

### 3.13 Pectoral Girdle and Fin (Figure 12)

**Figure 12:**
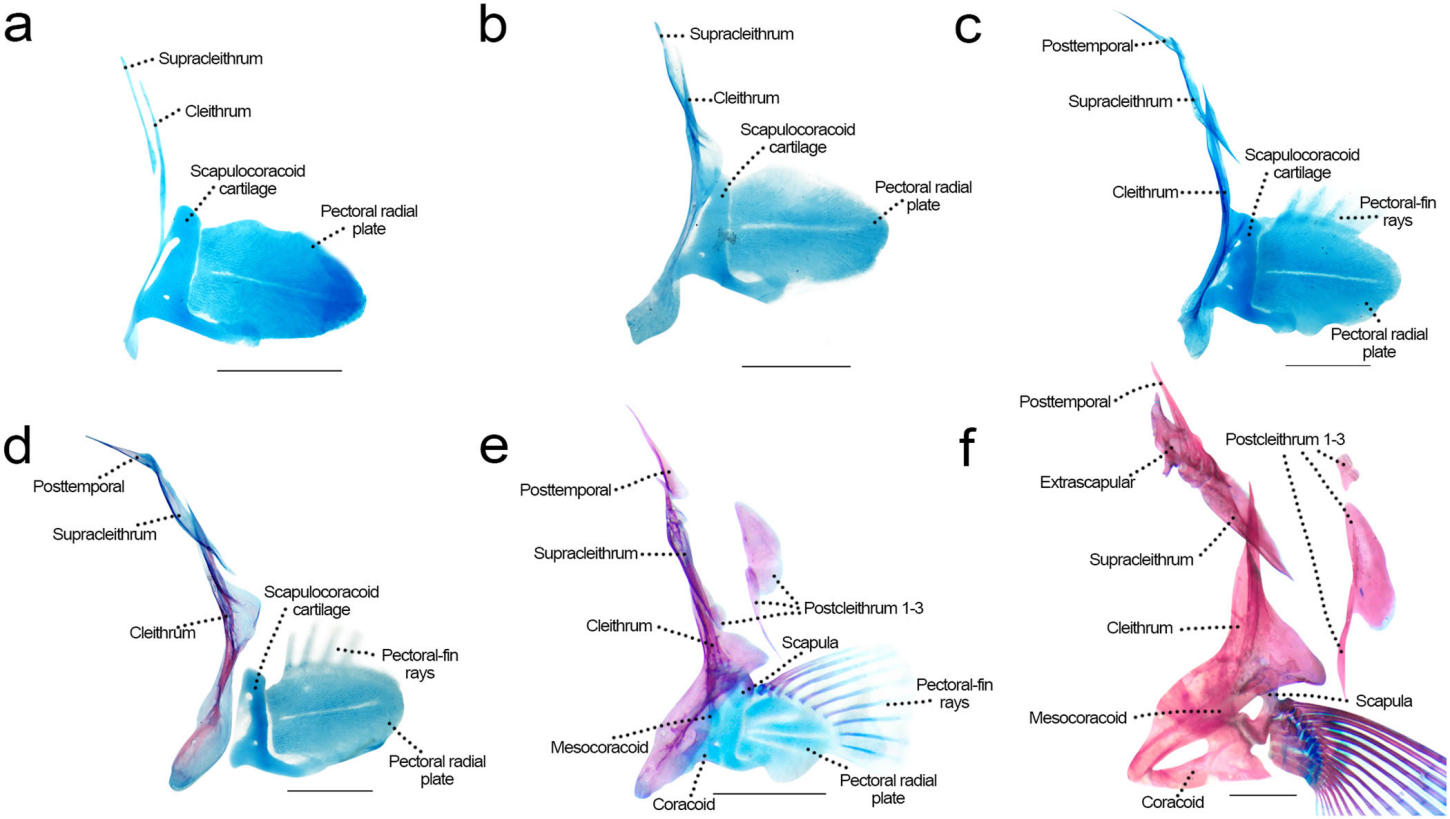
Pectoral girdle of *Leporinus oliveirai*, MZUEL 20844, in lateral view. (a) 9.0 mm SL. (b) 9.6 mm SL. (c) 11.0 mm SL. (d) 11.7 mm SL. (e) 15.7 mm SL. (f) 33.2 mm SL. Scale length: 1 mm.

Sequence of ossification: cleithrum – (supracleithrum + posttemporal) – postcleithrum 1–3 – (scapula + rays) – (coracoid + mesocoracoid) – extrascapular.

*Cleithrum*: first bone of body to appear at 5.1 mm NL, as a thin and elongated line, posterior to neurocranium. At 8.7 mm SL, its base is large and oval, and develops thickening. At 9.6 mm SL a posteriorly‐directed protuberance starts to develop at the middle of the cleithrum, to form the posterior process. The first signs of alizarin start at the middle of the bone, expanding to the borders, with bone fully ossified at 15.7 mm SL. The cleithrum is a large bone, with more or less rounded dorsal and ventral portions, and a distinctly triangular posterior process.

*Supracleithrum*: first visualized at 6.7 mm NL, thin and overlapping the dorsal tip of the cleithrum. At 9.6 mm SL the posterior margin grows distinctly, and 11.7 mm SL begins staining with alizarin, what led to the bone getting more elongate. The supracleithrum is fully ossified at 15.7 mm SL, sightly oval with pointy tips.

*Posttemporal*: small and thin bone articulating with the supracleithrum, first seen at 6.7 mm NL. The base gets slightly larger and the dorsal tip more elongate at 11.7 mm SL, when it starts to stain with alizarin, at 15.7 mm SL it is fully ossified, in the same shape. Posttemporal has two dorsally directed processes, one lateral and one medial.

*Postcleithra 1–3*: developing almost simultaneously, appearing at 12.5 mm SL, in the same shape as that of adults, growing and ossifying fast. Postcleithrum 1 is small and circular immediately posterior to where supracleithrum overlaps with cleithrum. Postcleithrum 2 is oval‐shaped, larger and longer than the former, posterior to cleithrum. Postcleithum 3 is a thin long line overlapping with postcleithrum 2, and medial to the fin‐rays.

*Scapula*: first appears at 11.0 mm SL, at the dorsal portion of the scapulocoracoid cartilage. Ossification starts at 13.6 mm SL, from posterior to anterior margin, being fully ossified at 27.2 mm SL, articulating with the first pectoral‐fin rays. The scapula forms a two‐armed bridge, and dorsally presents a process that expands posteriorly, and dorsally there is another triangular process.

*Coracoid*: starts to ossify at 15.7 mm SL, at the anteroventral portion of the scapulocoracoid cartilage, spreading quickly around the rest of the cartilage. At 27.2 mm SL, the only cartilaginous tip is the one connecting it to the cleithrum and the scapula. At 33.2 mm SL, the scapulocoracoid is mostly replaced, the coracoid presents a boomerang shape that becomes more robust and triangular in adult individuals.

*Mesocoracoid*: the mesocoracoid starts to ossify at 15.7 mm SL, medial to the scapulocoracoid cartilage, as a thin elongated cylinder, being fully ossified in juveniles (27.2 mm SL), when it has a similar shape to that in adults, i.e., a triangle with a large base that articulates with the scapula and coracoid and a thinner dorsal tip connected to the dorsalmost part of cleithrum.

*Extraexcapular*: first visualized in juveniles at 27.2 mm SL, when it is already ossified and similar to that of adults, small and oval with a triangular process on the dorsal portion.

*Pectoral‐fin rays*: the first three pectoral‐fin rays start to develop at 11.0 mm SL, associated with the dorsal portion of the pectoral radial plate at 12.3 mm SL two more rays develop and they grow in number as development progresses. The first rays can be seen stained with alizarin at 13.5 mm SL, from the base to the tip of each ray, with the most ventral rays ossifying later. All rays are fully ossified in juveniles (27.2 mm SL). Four pectoral radials, developed from cartilage and fully ossified only in juveniles (27.2 mm SL).

### 3.14 Pelvic Girdle and Fin (Figure 13)

**Figure 13:**
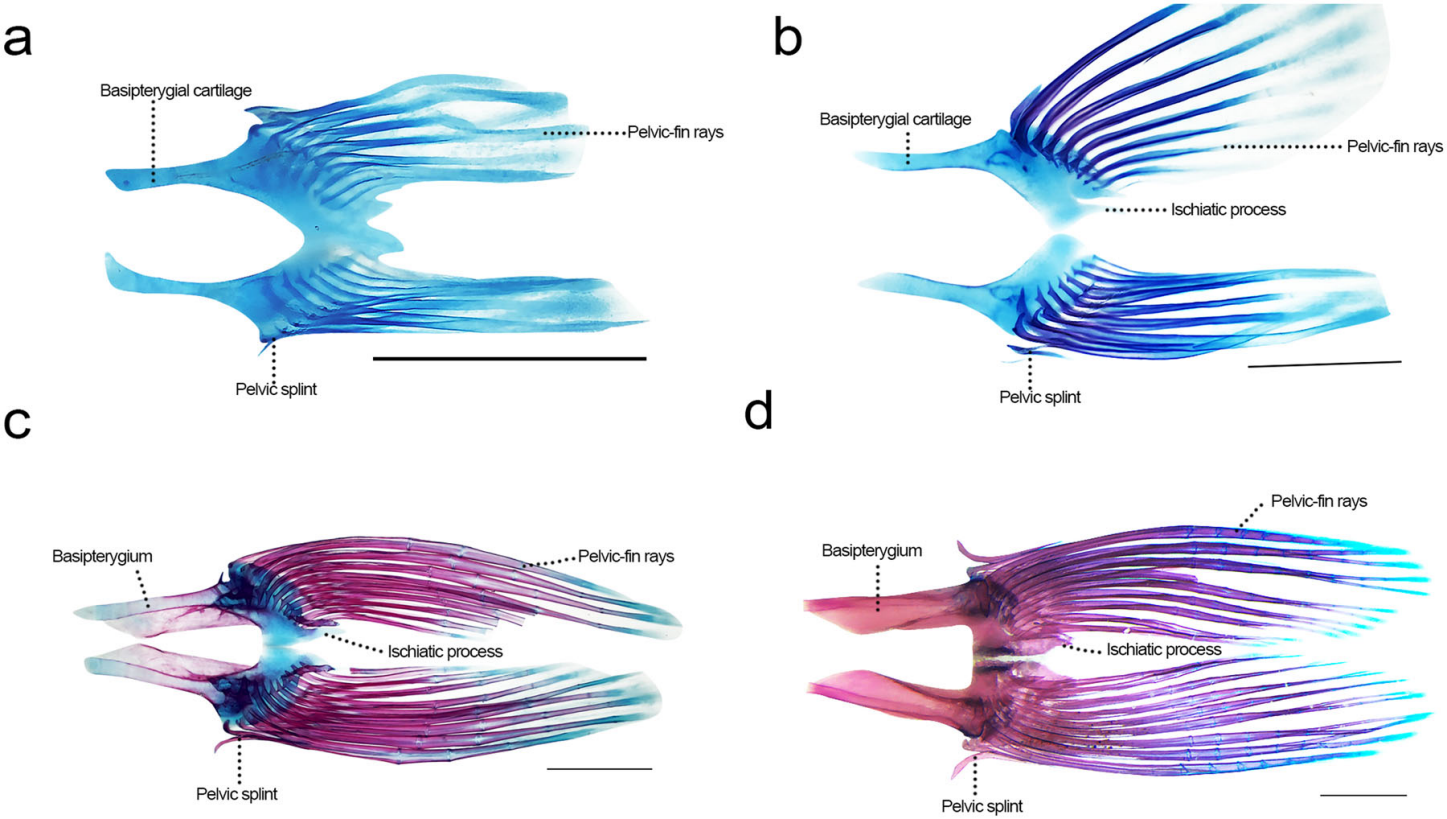
Pelvic girdle of *Leporinus oliveirai*, MZUEL 20844, in dorsal view. (a) 11.7 mm SL. (b) 12.1 mm SL. (c) 14.5 mm SL. (d) 33.2 mm SL. Scale length: 1 mm.

Sequence of ossification: (pelvic‐fin rays + basipterygium).

*Pelvic‐fin rays*: the nine pelvic‐fin rays plus pelvic splint appear at 11.0 mm SL and start to stain shortly after, at 11.8 mm SL. Ossification develops from anterior to posterior direction, finishing at 14.5 mm SL.

*Basipterygium*: the basipterygium cartilage appears at the same time as the rays, at 11.0 mm SL, with the ossification starting at 11.8 mm SL at the medial and lateral margins of the posterior portion of the bone. Ossification expands anteriorly and at 13.6 mm SL a thin layer of bone grows medially. The bone continues to develop, with the paired basipterygium getting more firmly connected to each other by cartilage at 14.5 mm SL. Complete ossification of the bone was only seen in juveniles (27.2 mm SL), the bone is long and thin on the anterior portion, and posteriorly its larger, ending with a prominent ischiatic process.

### 3.15 Dorsal Fin (Figure 14)

**Figure 14:**
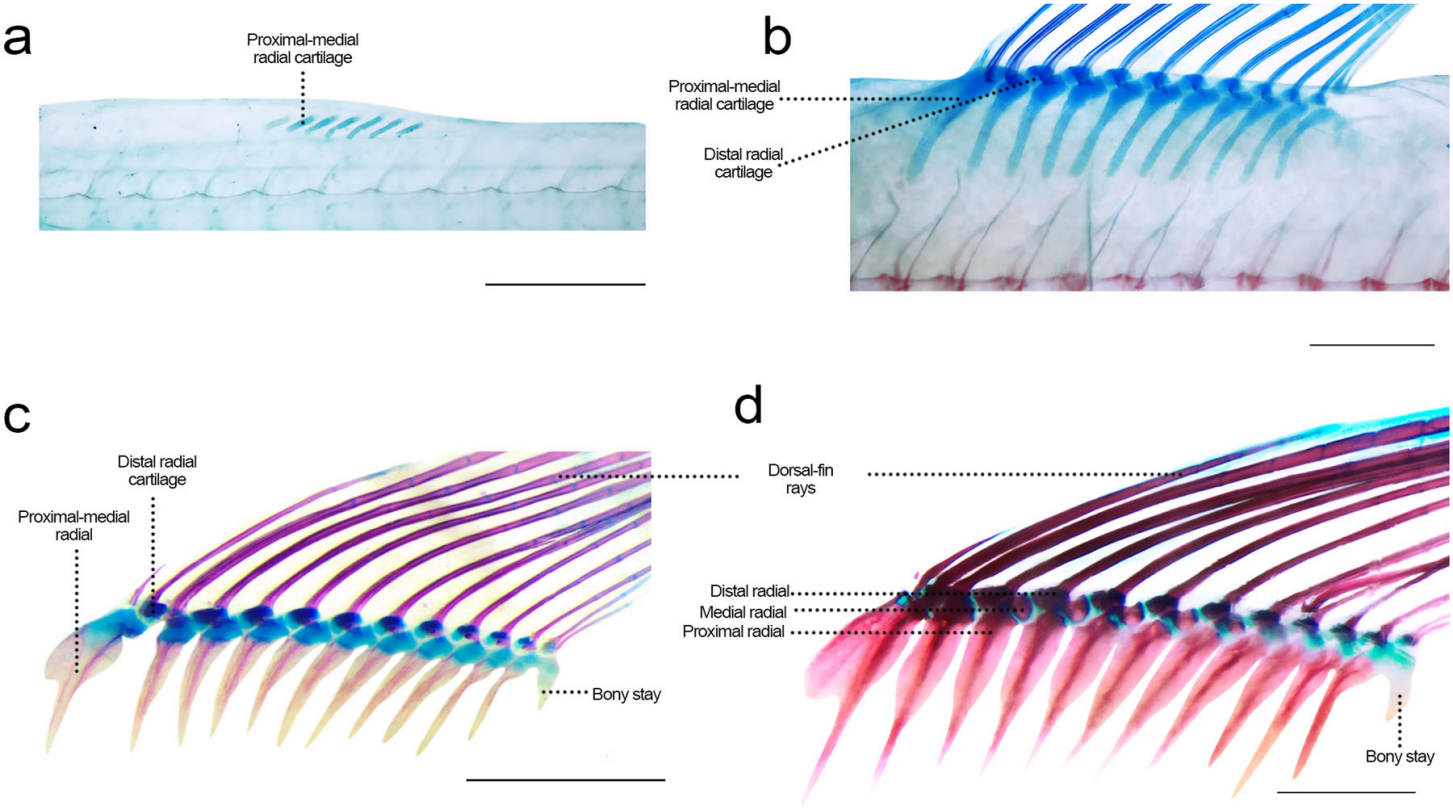
Dorsal fin of *Leporinus oliveirai*, MZUEL 20844, in lateral view. (a) 8.0 mm SL. (b) 11.7 mm SL. (c) 15.7 mm SL. (d) 27.2 mm SL. Scale length: 1 mm.

Sequence of ossification: rays – proximal‐medial radials – distal radials – bony stay.

*Dorsal‐fin rays*: the rays first appear at 8.7 mm SL as eleven elements (rays). Second to fourth ray start to ramify at 9.6 mm SL, and soon later the other rays start to ramify. Staining with alizarin starts at 11.0 mm SL on the anteriormost rays, and at 11.7 mm SL all rays are ossifying, completing it at 14.5 mm SL. The first ray greatly reduces in size in juveniles, becoming approximately half the size of the second ray; with both never ramifying.

*Proximal‐middle radials*: seven elements are observed at 8.0 mm SL, and finally eleven at 8.7 mm SL. Ossification starts at 11.8 mm SL, in the middle portion of the proximal‐middle radial cartilage, from the margins to the centre or each radial, circling the cartilage and expanding ventrally. At 16.3 mm SL, all radials are fully ossified except for the anterior extension of the first radial. In juveniles, at 27.2 mm SL, the division between proximal and middle radials is more evident. Despite starting development from the same cartilage precursors and ossifying concomitantly, all of the proximal and middle radials are two separate bones un juvenile and adult specimens.

*Distal radials*: appearing at 8.7 mm SL along with the dorsal‐fin rays and dorsal to the proximal‐middle radial cartilage, with ossification starting at around 16.3 mm SL. At 27.2 mm SL, ossification has finished on the first five radials (27.2 mm SL), and at 31.0 mm SL all distal radials are completely ossified.

*Dorsal‐fin bony stay*: observed at 10.3 mm SL as a small cartilaginous element posterior to the last proximal‐middle radial cartilage. It remains cartilaginous and with similar shape all through development.

### 3.16 Anal Fin (Figure 15)

**Figure 15:**
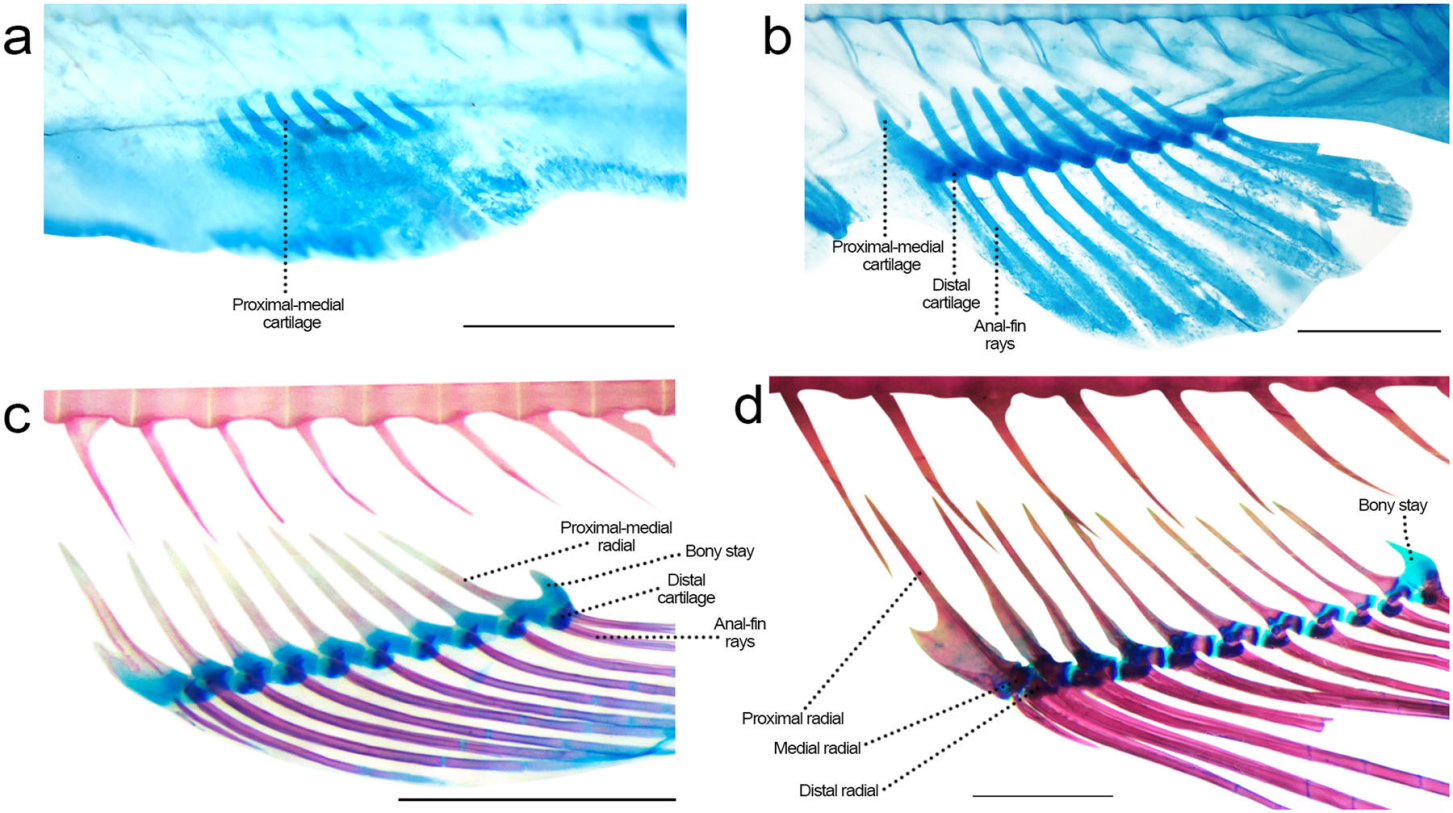
Anal fin of *Leporinus oliveirai*, MZUEL 20844, in lateral view. (a) 8.0 mm SL. (b) 9.8 mm SL. (c) 13.6 mm SL. (d) 31.0 mm SL. Scale length: 1 mm.

Sequence of ossification: rays – proximal‐medial radials – distal radials.

*Anal‐fin rays*: at 8.4 mm SL the anteriormost rays appear, at 8.7 mm SL ten cartilaginous rays are present, and at 9.6 mm SL ramification starts with middle rays, at the same time as the rays start to stain with alizarin, with completion at 14.5 mm SL.

*Proximal‐middle radials*: seven proximal‐middle radial cartilages observed at 7.3 mm NL, and ten at 8.4 mm SL, with the first one enlarged and likely encompassing a small proximal radial anterior to all ten separate elements. Ossification starts at 11.8 mm SL, in the middle portion of the cartilage, circling each radial, expanding dorsally, and by 16.3 mm SL elements are almost completely ossified. Despite starting development from the same cartilage precursors and ossifying concomitantly, all of the proximal and middle radials are two separate bones in juvenile and adult specimens. The first enlarged pteriophore bearing two unbranched rays in adults. The last element forming the anal‐fin stay.

*Distal radials*: appearing at 8.4 mm SL with the anal‐fin rays and dorsal to the proximal‐middle radial cartilage, with ossification starting at around 16.3 mm SL. At 27.2 mm SL, the anteriormost distal radials are ossified and at 31.0 mm SL all elements are completely ossified.

### 3.17 Caudal Skeleton and Fin (Figure 16)

**Figure 16:**
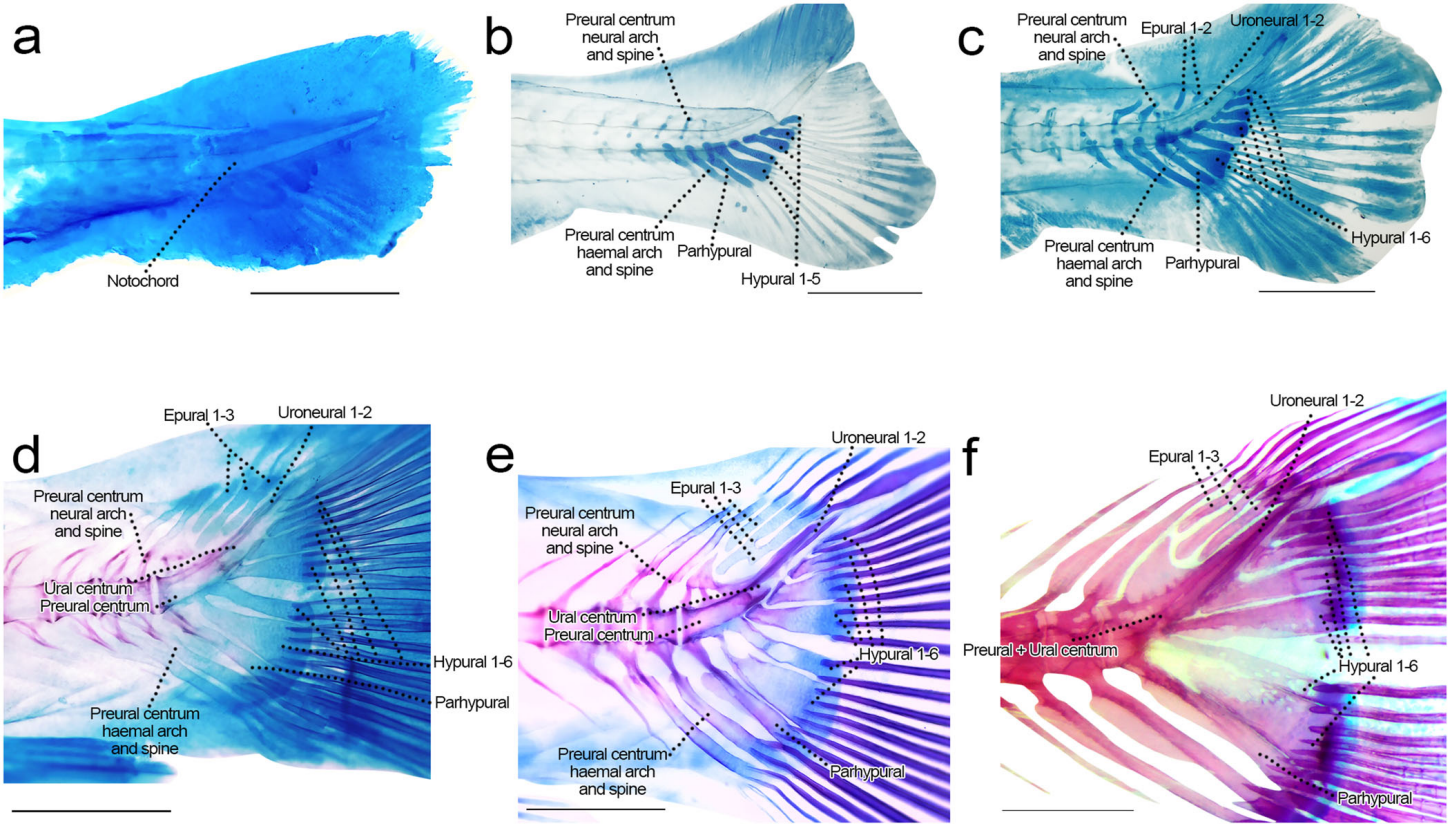
Caudal fin of *Leporinus oliveirai*, MZUEL 20844, in lateral view. (a) 6.4 mm SL. (b) 7.3 mm SL. (c) 8.0 mm SL. (d) 9.6 mm SL. (e) 11.7 mm SL. (f) 33.2 mm SL. Scale length: 1 mm.

Sequence of ossification: (hypural 1–6 + parhypural + haemal and neural arches PU2‐3 + uroneural 1–2 + preural centra 2–3 + [preural 1 + ural centra 1] + principal rays) – (haemal and neural spines PU2‐3 + procurrent rays) – epural 1–3.

*Principal caudal‐fin rays*: the first ones to develop are the ones associated with hypurals 2, 3 and 4 at 7.3 mm NL, and by 11.0 mm SL all rays appeared. Ramification starts at 9.8 mm SL, and progresses with the ossification to the posterior direction.

*Procurrent caudal‐fin rays*: both dorsal and ventral procurrent caudal‐fin rays start to develop at 8.7 mm SL, four ventral and five dorsal, the ones closer to the principal rays ossifying first, and by 12.1 mm SL all rays are ossified. In juveniles there's five ventral rays and six dorsal rays.

*Hypurals 1‐6*: the hypurals cartilage begin development at 6.4 mm NL, and initiate ossification chondrally at 9.6 mm SL, simultaneously in all elements, from the anterior tips, connected to the notochord, to the posterior margin where caudal‐fin rays are connected. At 11.8 mm SL, hypurals 4–6 are almost completely ossified, finishing at 12.2 mm SL. At 13.6 mm SL all hypurals are completely ossified except for their posterior margins, just like in the adults.

*Parhypural*: starts to ossify chondrally at 9.6 mm SL in the anteriormost margin of the parhypural cartilage, ossification expanding ventrally at 11.0 mm SL, until it is fully ossified at 11.8 mm SL. As in hypurals, the posterior margin of parhypural remains cartilaginous all through development. At 11.0 mm SL a semi‐circular anterior projection starts to develop, becoming oval shape, similar to that of adults in 12.5 mm SL.

*Neural arches and spines of pre‐ural centra 2 and 3*: the cartilage appears at 7.3 mm NL and is already well developed at 8.0 mm SL, with the chondral ossification starting at 9.6 mm SL on the arches, which are fully ossified at 11.7 mm SL. The neural spines start to ossify at 11.0 mm SL around the cartilaginous precursors, becoming fully ossified at 12.2 mm SL except for the posteriormost margin.

*Haemal arches and spines of pre‐ural centra 2 and 3*: the cartilages were observed at 6.6 mm NL, with ossification starting chondrally on the arches at 9.6 mm SL and finishing at 11.7 mm SL. At 11.0 mm SL the ossification starts around the cartilage spines, with the arches fully ossified at 12.2 mm SL, when only the spine tips are cartilaginous.

*Pre‐ural centra 2 and 3*: both appear at 8.0 mm SL, and are distinctly separated at 8.7 mm SL, anterior to the parhypural. The ossification is similar to what was described to other vertebral centra, starting at 9.6 mm SL and finishing at 11.7 mm SL.

*Pre‐ural centra 1 and ural centra*: the pre‐ural centra 1 and ural centra 1 are two distinct structures at the beginning of development, and the chondral ossification starts simultaneously at 9.6 mm SL. The pre‐ural centra 1 is associated with the parhypural, hypural 1 and 2, and the ural centra 1 with hypural 3. At 11.0 mm SL both centra appear fused, with no gap in‐between, forming the compound centrum, which is ossified by 11.7 mm SL. After the fusion, the centrum stays rectangular and elongated until the juvenile stage, where its oval and restricted to the most anterior portion of the hypural plate.

*Uroneurals*: both uroneurals were observed at 8.0 mm SL, as thin and long bones immediately dorsal to the notochord, and hypurals 2–4. At 11.0 mm SL, uroneural 1 is elongated and extended from hypural 2 to 6, and fused to the compound centra. At the same time, uroneural 2 is more posterior, extended from hypurals 4 to 6.

*Epurals*: epurals 1 and 2 cartilaginous elements appear at 8.0 mm SL, and epural 3 at 8.7 mm SL, dorsal to the developing compound centrum and uroneurals. The chondral ossification starting simultaneously on the three thing and elongated elements at 12.2 mm SL.

## 4 Discussion

### 4.1 Developmental Sequence

In general, it is possible to say that the development of *Leporinus oliveirai* is similar to that previously described for other Characiformes (i.e., *Salminus brasiliensis*, *Makunaima pittieri, Brycon moorei*, *Bario sanctaefilomenae*, and *Prochilodus argenteus*). The most notable changes occur in the head, where the most relevant phylogenetic characters of the family are associated with the variation in jaw morphology (Sidlauskas and Vari 2008). After hatching, *L. oliveirai* is boneless at 3.8 mm NL, and specimens ranging from 4.6 mm NL to 4.9 mm NL (i.e., immediately post‐hatching or first dph) already showed the cartilages of the branchial apparatus and the pectoral bud. The first bones to appear (at 5.1 mm NL, two dph) are the cleithrum and the upper and lower jaw bones (Table 1, Appendix 2), followed by the branchiostegal rays, urohyal, supracleithrum and posttemporal (6.0 mm NL to 6.5 mm NL). The appearance of these first elements is likely related to the beginning of exogenous feeding. The sequence is similar from what was described to other characiforms with slight variation in the order of the same bones, especially regarding the early appearance of the jaws and teeth (Vandewalle et al. 2005; Walter 2012; Mattox et al. 2014; Carvalho and Vari 2015; Marinho 2022). Nonetheless, the pharyngeal jaws and the suspensorium bones appear slightly later (around 9 mm SL) in *L. oliveirai* than in other Characiformes. Additionally, in *Salminus brasiliensis* and *Prochilodus argenteus*, the anguloarticular do not start to ossify concomitantly to the other jaw bones as observed in *L. oliveirai* or almost at the same time as in *Makunaima pittieri*.

Other notable differences are the early appearance of the pharyngeal tooth plates, ceratohyal, quadrate, hyomandibular and ceratobranchials in *S. brasiliensis* and *M. pittieri*, while in *L. oliveirai* those bones develop much later.

The palatine cartilage was described as an anterior process of the palatoquadrate cartilage in other Characiformes, extending in the direction of the ethmoid plate (Walter 2012; Mattox et al. 2014; Carvalho and Vari 2015; Marinho 2022). In *L. oliveirai*, it was possible to see the cartilage for the first time at 5.6 mm NL, with an oval and independent cartilage between it and the ethmoid plate. The palatine cartilage is straight in the direction of the snout, becoming curved anteriorly during the development, until it reaches the shape of the adult (Ito et al. 2023, fig. 4). As discussed by Sidlauskas and Vari (2008) and Sidlauskas et al. (2022), the common condition for Anostomidae is the presence of this curved autopalatine process, its absence being an important synapomorphy of the subfamily Anostominae, and *Leporellus*.

The formation of the quadrate‐metapterygoid fenestra in fishes was recently discussed in detail by Britz et al. (2023). The authors point out that Characiformes not only notably present the fenestra, but it is also present from the beginning of the development, already in the palatoquadrate cartilage. Noteworthy, two characiforms in their study do not follow the pattern, *Lebiasina* (in which it forms later in development) and *Pyrrhulina* (in which the fenestra is never formed). Regardless, *Leporinus oliveirai* conforms to the pattern observed across Characiformes, with the fenestra in the palatoquadrate cartilage being visible at 5.0 mm NL (2dph).

Many of the neurocranium and caudal‐skeleton elements started to ossify simultaneously in our studied specimens, most at 9.6 mm SL. Noteworthily, only 9.6 mm SL or larger specimens were stained with alizarin. Nevertheless, it is likely that the timing of the ossification of some of these elements may be slightly different, and we could not notice that due to limited samples at that size range. Cubbage and Mabee (1996), in their description of the skull and paired fins of *Danio rerio*, discussed that not necessarily all specimens of the same size will present the same condition due to a slight variation in the timing of ossification. Subsequent ontogeny works followed this idea (Bird and Mabee 2003; Mattox et al. 2014; Marinho 2022), stating the smallest size the presence of a bone was registered, and the size in which all specimens where ossification was present (fixed presence). The absence of this variation in our study is likely a matter of a small sample rather than a lack of existing variation.

The last bones to appear in *L. oliveirai* are the infraorbitals 4, 5 and 6, extrascapular, autopalatine, fins radials, and sclerotic. In fact, only the infraorbitals 1 to 3 could be seen shortly before the juvenile stage. A similar pattern was observed in *Makunaima pittieri* (Marinho 2022) and in *Salminus brasiliensis* (Mattox et al. 2014). However, it is important to note that *M. pittieri* does not present a supraorbital bone. In both *L. oliveirai* and *S. brasiliensis*, the sequence is sightly stair‐like, even with our study presenting more concomitant ossifications, while in *M. pittieri* there is a discrepancy of around 15 mm between infraorbital 3 and rhinosphenoid (not present in the other two species).

In juveniles (27.2 mm SL and 33.2 mm SL), almost all the bones look the same as in adult individuals (MZUSP119371, 103.6 mm SL) of *L. oliverai* (see also Ito et al. 2023), with a few exceptions, mostly related to their relative size and shape. The dorsal tip of the premaxilla becomes more curved posteriorly in adults, and the mouth position is terminal (or sightly superior) in juveniles whereas in the adult it is distinctly subterminal. The skull looks much more compressed laterally in adults. The snout is longer, with the fontanelle opening smaller, the parasphenoid posterior processes expanding posteriorly, and the mesethmoid and vomer more prominent ventrally. The metapterygoid‐quadrate window becomes smaller as both bones acquire a more distinctly triangular shape, and the cartilage portions where the bones connect are less distinct. In the caudal fin, the dorsal process of the neural complex becomes more rectangular, the dorsoposterior margin maintains the curvature, but it is smaller than in juveniles.

### 4.2 Jaw Teeth Development

Anostomid jaw teeth are unique among Characiformes for having large incisiform teeth with cups or with blunt cutting edges aligned in a single row and weakly implemented on the posterior face of the jaws (pleurodont) (Myers 1950; Garavello and Britski 2003). In addition, all anostomids have only a few, (one to five) teeth on each premaxilla and dentary (Sidlauskas and Vari 2008; Britski and Birindelli 2008). These features are so unique that the comparisons among other Characiformes are puzzling (Vari 1983; Sidlauskas and Vari 2008). Anostomidae is closely related to a clade containing Chilodontidae, Prochilodontidae, and Curimatidae (Melo et al. 2021). Prochilodontidae present multiple functional villiform teeth attached to the lips (Roberts 1973; Guisande et al. 2012), while Curimatidae only present teeth during the larval stage (Géry 1977: figs. on pages 231 and 234), in shape and disposition similar to the one seen in larvae of *Leporinus oliveirai*.

While a few studies have focused on Anostomidae ontogeny, only the PhD dissertation of Giovannetti (2019) analysed the development of teeth. The author analysed five unvouchered larval specimens identified as *Leporinus* sp. and reported the presence of multiple conical teeth on both the premaxilla and dentary during early stages. However, the author made no further comments regarding the implications of this characteristic or illustrated this feature on the specimens.

Conical acrodont teeth is the most basic condition when it comes to Actinopterygii fish (Atukorala and Franz‐Odendaal 2014), including Characiformes, whereas in anostomids, four teeth on the premaxilla is the condition found on most species (Sidlauskas and Vari 2008). The presence of multiple acrodont and conical teeth in anostomid larvae is therefore the plesiomorphic condition for this character, whereas its replacement by pleurodont incisiform teeth is an apomorphic condition, an outstanding synapomorphy for the Anostomidae.

Furthermore, the most common condition in Anostomidae is to have four teeth on each premaxilla and dentary. Nevertheless, in several cases (see Myers 1950; Britski and Birindelli 2008; Ramirez et al. 2017), there are species (or genera) characterized by having only three teeth on each premaxilla or dentary (or on both). This condition is even more extreme in *Gnathodolus bidens* Myers 1927, in which only a single enlarged tooth is present on each dentary (Myers 1927; Winterbottom 1980). Our study shows us that four teeth on both jaws may be the plesiomorphic condition, and a reduction of that number might be derived, and likely tracible through ontogeny. A similar case was described for *Astyanax mexicanus* (De Filippe 1853), which was found to have three tooth replacements during larvae development, the first one being conical teeth, and the last one being multicuspid as in most adult tetras (Atukorala and Franz‐Odendaal 2014).

Additional studies, especially those including histological and genetic techniques are still necessary for a better understanding of the implications on how this shift in the dentition is correlated to the evolution of Anostomidae and also Characiformes, as it has been extensively done with *Danio rerio*, for which we have identified the genes that affect the development of certain bones including those linked to the teeth formation (Mabee et al. 2007). In that direction, Sousa (2014) used markers to identify the expression of bone morphogenetic proteins (BMP) 2 and 4 on the mouth region of the larvae of *Megaleporinus obtusidens*. However, the genetic mechanisms and the role of BMP2 and BMP4 developing of oral and pharyngeal jaw teeth are still unclear (Atukorala and Franz‐Odendaal 2014).

### 4.3 Jaw Bones Development and Shape

The jaw bones are among the first bony elements to appear in Characiformes and quickly become fully ossified. In *Salminus brasiliensis*, the dentary and the premaxilla can be first seen at 5.1 mm NL and are fully formed at 12.7 mm SL and 18.4 mm SL, respectively, with multiple canine and conical teeth in two rows (Mattox et al. 2014). Vandewalle et al. (2005) found a similar development and shape pattern in *Brycon moorei*, describing the development of the skull based on hours post‐hatching. Species of both aforementioned genera, *Salminus* and *Brycon*, possesses conical teeth on the maxilla, and all three bones are much longer than those of *L. oliveirai*, extending posteriorly and being ventral to the anterior portion of the hyopalatine arch (Mattox et al. 2014). On the other hand, the jaws in *L. oliveirai* do not grow considerable in size during development, being small and thicker in juveniles and adult individuals.

In *Makunaima pittieri*, development starts even earlier, at 3.8 mm NL for the dentary and 4.0 mm NL for the premaxilla, when small conical teeth develop (Marinho 2022). Unlike those described for *Salminus*, in *M. pittieri*, the conical teeth are replaced by cuspidated teeth, with the premaxilla bearing two rows of teeth, and the maxilla having conical and cuspidated teeth. In the latter, all three jaw bones are more similar in shape to those of *L. oliveirai*, although they also extend ventrally to the anteriormost portion of the hyopalatine arch (Marinho 2022). In contrast, the jaws are directly anterior to it in *L. oliveirai*. Walter (2012) found similar results with *Bario sanctaefilomenae*, but notably the maxilla only presents a single incisiform tooth.

Another characiform with a detailed osteological description of the head is *Prochilodus argenteus*, with multiple conical teeth appearing as early as three dph, which are replaced by flattened functional teeth attached to the lips in adults (Carvalho and Vari 2015). In *Prochilodus* the premaxilla and maxilla are relatively small during their development. The dentary is longer during the early stages but gets restricted to the anterior portion of the head during development, similar to *L. oliveirai*.

As previously mentioned, Curimatidae presents teeth only during the larval stage, similar to the pattern *L. oliveirai* presented. Nevertheless, the shape and position of the bones are considerably different, and differ little during the development, as illustrated by Géry (1977: 231). *Curimata* presents an elongated premaxilla, sightly rectangular and rather thin, almost horizontal. The maxilla articulates with the posterior tip of the premaxilla, being a little less horizontal, with the dorsal tip slightly curving in dorsal direction. It has an overall tear shape, with the anterior margin thinner than the larger posterior margin and is toothless even in early development. The lower jaw is triangular and quite similar to Anostomidae, except the teeth appear on the anterior margin instead of dorsally, almost vertical to the dentary. The other drawing, identified as possibly *C. helleri*, has the same shape and disposition overall, with the anteroventral tip of the dentary curving ventrally (Géry 1977: 234).

We are unable to answer if the condition in which the maxilla is not articulating with the jaws is a consequence of clearing and staining procedures or if the bone is indeed just attached to a thin layer of skin. Machado‐Evangelista et al. (2015) analysed the ontogenetic shifts related to the diet of *Leporinus reticulatus*, however, the smallest specimen they cleared and stained was 24.0 mm SL. Still, their study shows that younger *Leporinus* have a more animal‐based diet, shifting more towards herbivory as the fish grows and change the teeth morphology (tricuspid then one cuspid) and mouth position (terminal to subterminal), similar to the pattern seeing in *L. oliveirai*. The teeth morphology can indicate that larvae in the early stages follow this pattern of eating from animal sources, however, more detailed studies are needed to understand the role of the maxilla in the articulation of the jaws in those stages.

### 4.4 Fusion of the Fourth and Fifth Infraorbital

In Anostomidae and closely related families, and also in Characiformes as a whole, the most common condition is for the fourth and fifth infraorbital to be two separate bones (Sidlauskas and Vari 2008; Mirande 2010). In Anostomidae, the fusion of these two elements were reported in a few species, and was considered a derived characteristic (Sidlauskas and Vari 2008; Birindelli and Britski 2009).

For the original description of *Leporinus oliverai*, two adult specimens (MZUSP119371) were cleared and stained: one has the infraorbitals 4 and 5 separate (Ito et al. 2023: fig. 2), while the other presents the two bones fused. This characteristic was treated as an anomaly of this particular specimen therein, considering the common condition for those two bones in the group.

The infraorbital series is one of the latest components of the skeleton to develop in *L. oliveirai*, at around 15.7 mm SL, with the infraorbitals 4 to 6 being between the last bones to start developing, only seen in juveniles. In all examined juveniles herein studied (27.2 mm SL to 33.2 mm SL), the fourth and fifth infraorbital were present from the beginning as a single element. Considering one of the adult specimens also presents the two bones as a single element, it is more likely to presume the *L. oliveirai* does not present separate infraorbitals 4 and 5. The specimen used in the original description possible presented an anomaly.

Considering the difference in size of the juveniles and the adult specimens and the late development of those bones, while less likely, it is possible to speculate that the infraorbitals 4 and 5 initially appear as a single element and much later become separated, meaning the original description is correct. With current data we are unable to answer those speculations, being necessary more specimens to analyse the skeleton of both juveniles and adults.

## 5 Conclusion

Our study is the first to describe the complete skeletal ontogeny of an anostomid and the third of a characiform, providing essential data for understanding the evolution of fishes, common patterns and differentiation during development. The unique pattern of the jaws and tooth development of *L. oliveirai* showed details that help unravel the evolutionary history of characters that are unique to the Anostomidae, and therefore, the evolution of this family among Characiformes. Nevertheless, further research is necessary for a complete understanding of the development, homology, and evolution of the bones in Anostomidae.

## Author Contributions

**Mariana Pascoal Boaretto:** conceptualization, investigation, writing – original draft, methodology, writing – review and editing, formal analysis, data curation. **Marcos Venturieri:** investigation, methodology, data curation, writing – original draft. **José Luís Olivan Birindelli:** conceptualization, funding acquisition, writing – original draft, writing – review and editing, visualization, validation, methodology, supervision.

### 1 Peer Review

The peer review history for this article is available at https://www.webofscience.com/api/gateway/wos/peer-review/10.1002/jmor.70053.

## Supporting information

Appendix 1.

Appendix 2.

## Data Availability

The data that supports the findings of this study are available in the Supporting Information of this article.
