# Supplementary material for: Ontogeny of the Skeleton of Leporinus oliveirai (Characiformes, Anotomidae)
Source: J Morphol. 2025 May 21;286(5):e70053. doi: 10.1002/jmor.70053 (PMC12093931; doi:10.1002/jmor.70053)
Supplement: Supplementary file 1 — Appendix 1. [file JMOR-286-e70053-s001.docx]

**Appendix 1.** Size (mm), age (days post-hatching) and life period of examined specimens of *Leporinus oliveirai*, MZUEL 20844, n=63. Notochord length (NL) was measured from the tip of the snout until the end of the notochord in specimens on yolk-sac and pre-flexion stages. Standard length (SL) was measured from the tip of the snout until the end of the hypural plate in specimens on flexion, post-flexion and juvenile stages. Specimens from Larval stages were classified according to Nakatani *et al*. (2001), with juveniles being considered only specimens with full complement of the fin rays.

| Size (mm) | Age (days) | Life Period |
| --- | --- | --- |
| 3.81 | 0 | Yolk sac |
| 4.57 | 1 | Yolk sac |
| 4.61 | 0 | Yolk sac |
| 4.68 | 1 | Yolk sac |
| 4.71 | 1 | Yolk sac |
| 4.89 | 0 | Yolk sac |
| 5.09 | 2 | Pre-flexion |
| 5.14 | 2 | Pre-flexion |
| 5.19 | 5 | Pre-flexion |
| 5.21 | 1 | Yolk sac |
| 5.27 | 3 | Pre-flexion |
| 5.30 | 2 | Pre-flexion |
| 5.37 | 4 | Pre-flexion |
| 5.43 | 4 | Pre-flexion |
| 5.50 | 3 | Pre-flexion |
| 5.51 | 4 | Pre-flexion |
| 5.55 | 7 | Pre-flexion |
| 5.59 | 5 | Pre-flexion |
| 5.60 | 5 | Pre-flexion |
| 5.66 | 8 | Pre-flexion |
| 5.77 | 6 | Pre-flexion |
| 5.93 | 6 | Pre-flexion |
| 6.04 | 13 | Pre-flexion |
| 6.05 | 5 | Pre-flexion |
| 6.06 | 7 | Pre-flexion |
| 6.08 | 6 | Pre-flexion |
| 6.22 | 14 | Pre-flexion |
| 6.27 | 10 | Flexion |
| 6.35 | 12 | Pre-flexion |
| 6.38 | 13 | Pre-flexion |
| 6.43 | 11 | Pre-flexion |
| 6.52 | 10 | Flexion |
| 6.64 | 15 | Pre-flexion |
| 6.66 | 12 | Pre-flexion |
| 6.68 | 7 | Flexion |
| 6.73 | 15 | Pre-flexion |
| 6.82 | 14 | Flexion |
| 7.35 | 16 | Flexion |
| 7.98 | 16 | Flexion |
| 8.38 | 20 | Flexion |
| 8.53 | 9 | Flexion |
| 8.66 | 20 | Flexion |
| 8.98 | 9 | Flexion |
| 9.63 | 15 | Flexion |
| 9.80 | 15 | Flexion |
| 9.97 | 12 | Flexion |
| 10.34 | 12 | Flexion |
| 10.98 | 30 | Post-flexion |
| 11.69 | 20 | Post-flexion |
| 11.79 | 25 | Post-flexion |
| 12.14 | 25 | Post-flexion |
| 12.20 | 25 | Post-flexion |
| 12.33 | 25 | Post-flexion |
| 12.52 | 30 | Post-flexion |
| 13.18 | 20 | Post-flexion |
| 13.56 | 30 | Post-flexion |
| 14.53 | 30 | Post-flexion |
| 15.76 | 30 | Post-flexion |
| 16.30 | 45 | Post-flexion |
| 27.18 | 60 | Juvenile |
| 29.56 | 60 | Juvenile |
| 31.00 | 60 | Juvenile |
| 33.19 | 60 | Juvenile |
